# GADMA2: more efficient and flexible demographic inference from genetic data

**DOI:** 10.1093/gigascience/giad059

**Published:** 2023-08-23

**Authors:** Ekaterina Noskova, Nikita Abramov, Stanislav Iliutkin, Anton Sidorin, Pavel Dobrynin, Vladimir I Ulyantsev

**Affiliations:** Computer Technologies Laboratory, ITMO University, St. Petersburg 197101, Russia; HSE University, St. Petersburg 194100, Russia; Computer Technologies Laboratory, ITMO University, St. Petersburg 197101, Russia; Laboratory of Biochemical Genetics, St. Petersburg State University, St. Petersburg 199034, Russia; Computer Technologies Laboratory, ITMO University, St. Petersburg 197101, Russia; Human Genetics Laboratory, Vavilov Institute of General Genetics RAS, Moscow 119991, Russia; Computer Technologies Laboratory, ITMO University, St. Petersburg 197101, Russia

**Keywords:** demographic inference, population genetics, genetic algorithm, hyperparameter optimization

## Abstract

**Background:**

Inference of complex demographic histories is a source of information about events that happened in the past of studied populations. Existing methods for demographic inference typically require input from the researcher in the form of a parameterized model. With an increased variety of methods and tools, each with its own interface, the model specification becomes tedious and error-prone. Moreover, optimization algorithms used to find model parameters sometimes turn out to be inefficient, for instance, by being not properly tuned or highly dependent on a user-provided initialization. The open-source software GADMA addresses these problems, providing automatic demographic inference. It proposes a common interface for several likelihood engines and provides global parameters optimization based on a genetic algorithm.

**Results:**

Here, we introduce the new GADMA2 software and provide a detailed description of the added and expanded features. It has a renovated core code base, new likelihood engines, an updated optimization algorithm, and a flexible setup for automatic model construction. We provide a full overview of GADMA2 enhancements, compare the performance of supported likelihood engines on simulated data, and demonstrate an example of GADMA2 usage on 2 empirical datasets.

**Conclusions:**

We demonstrate the better performance of a genetic algorithm in GADMA2 by comparing it to the initial version and other existing optimization approaches. Our experiments on simulated data indicate that GADMA2’s likelihood engines are able to provide accurate estimations of demographic parameters even for misspecified models. We improve model parameters for 2 empirical datasets of inbred species.

## Introduction

The evolutionary forces form a genetic variety of closely related species and populations. Principal historical events like divergence, population size change, migration, and selection could be reconstructed from the genetic data using different algorithmic and statistical approaches. Inference of complex demographic histories is widely applied in conservation biology studies to identify major events in the population’s past [1–3]. It supplements archaeological information about the historical processes that have left no paleontological records. Finally, demographic histories form the basis for subsequent population studies and medical genetic research.

In recent years, many methods for demographic inference have appeared to investigate the demographic histories of species or populations [4–12]. Some of them give a point estimate for the unknown demographic parameters [4, 7, 11] while others give the distribution thereof [5, 6, 8]. In this article, we focus inclusively on the former. Most methods that provide point estimates consist of 2 independent components. The first component provides means to compute data statistics under a proposed demographic history and compare them with real data by the log-likelihood value. One of the most widely used data statistics is the allele frequency spectrum (e.g., [4, 7, 10]). However, newer methods based on 2-locus [13] and linkage disequilibrium (LD) statistics [14, 15] have also become available. This article will denote the first component as an *likelihood engine*. The second component of existing tools is *optimization*. It requires a user-defined model of demographic history and performs a search of the maximal likelihood model parameters using different optimization algorithms. While a number of optimization techniques are provided, they often turn out to be ineffective in practical applications [16].

In 2020, we presented a new software GADMA [16] for unsupervised demographic inference from the allele frequency spectrum (AFS) data. It provides a common interface for various already existing likelihood engines and introduces new global search optimization based on a genetic algorithm. GADMA does not require complicated model specification. Instead, it takes *model structure* that determines how many time epochs are included in the model. Previously, models of demographic history were parameterized only by continuous parameters and had fixed population size dynamics. Constant size or exponential growth could be examples of such dynamics. GADMA’s model with structure extends the regular concept of a model by including dynamics as discrete model parameters. Thus, it can automatically construct history as a sequence of time epochs with desired parameter types from blocks of constant, linear, and exponential size changes. The researcher has control over the types of model parameters to infer. For example, all migrations could be disabled.

It was shown that the proposed genetic algorithm approach in GADMA has better performance than previously existing optimization algorithms both on simulated and real datasets [16]. GADMA proved itself capable of finding demographic histories that attain higher log-likelihood than the histories reported in the literature and obtained using the default optimization routines of ∂a∂i or *moments*. Moreover, using demographic models with structure, GADMA was able to find a new demographic history of modern human populations that is both paleontologically plausible and has better log-likelihood than the existing “Out-of-Africa” scenario from Gutenkunst et al. [4]. Since its initial publication, GADMA has been applied in several studies on a variety of species: Xiong et al. [17], Valdez and D’Elía [18], Pazhenkova and Lukhtanov [19], Cassin-Sackett et al. [20], and Buggiotti [21].

The initial version of GADMA features only 2 likelihood engines: ∂a∂i [4] and *moments* [7]. Both of these engines compute the allele frequency spectrum statistics using the Wright–Fisher diffusion-based approach and thus provide similar results. Among the variety of other available tools, we can highlight methods based either on AFS (*momi2, fastsimcoal2*), LD statistics (*momentsLD*), or haplotype data (diCal2) as potential additions to the supported engines in GADMA. Some already implemented features of ∂a∂i and *moments*, like the inference of selection and dominance rates, are not included in the first version of GADMA. Both ∂a∂i and *moments* have been upgraded since these programs were first published and since GADMA’s initial release. For example, ∂a∂i introduced inference of the inbreeding coefficients [22], started to support demographic histories involving 4 and 5 populations and enabled graphics processing unit support [23]. In light of these advancements, we have sought to extend GADMA in several directions to support new features and engines and further enhance its optimization algorithm.

In this article, we describe new capabilities implemented in GADMA2. We compare supported likelihood engines of GADMA2 on 2 simulated datasets for different demographic models. Furthermore, we demonstrate the efficiency of the updated version on 2 empirical datasets of inbred species from Blischak et al. [22].

**Figure 1:**
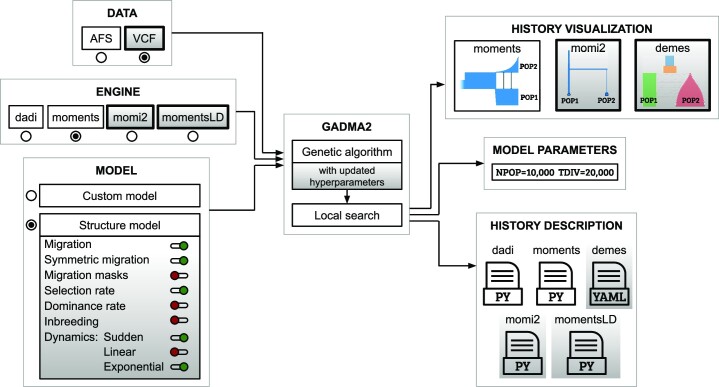
Scheme of GADMA2. New features and enhancements are marked with a gradient gray color. GADMA2 takes input genetic data presented in either AFS or VCF formats, engine name, and model specifications and provides inferred model parameters, visualization, and descriptions of the final demographic history.

GADMA2 has an updated core codebase and implements a more efficient and flexible unsupervised demographic inference method. The improved version extends the initial GADMA in several ways (Fig. 1). First, the genetic algorithm in GADMA2 is improved by hyperparameter optimization. New values of the genetic algorithm hyperparameters that provide more efficient and stable convergence are found. Second, GADMA2 provides more flexible control of the model specification for automatic model construction. For example, it is possible to include inferences about selection and inbreeding coefficients. Third, 2 new likelihood engines are integrated: *momi2* and *momentsLD*. Thus, GADMA2 supports 4 engines overall. Lastly, several functional enhancements are integrated, including the ability to use data in variant call format (VCF) format and new engines for history representation and visualization (*momi2* and *demes*).

## Materials and Methods

### Datasets

We use several datasets in this work. Datasets for the hyperparameter optimization are taken from the Python package deminf_data v1.0.0 (Supplementary Fig. S1) that is available on GitHub via the link [24]. Deminf_data contains various datasets with both real and simulated AFS data. Simulations are performed with *moments* [7] software. Each dataset is named according to the convention described in Supplementary Fig. S1 and includes (i) the allele frequency spectrum data, (ii) the model of the demographic history, and (iii) bounds of the model parameters. Full descriptions of the data and demographic model parameters of datasets are available in the repository on GitHub. For hyperparameter optimization, we used 10 datasets from deminf_data: 4 as training problem instances and 6 for testing. Basic descriptions of these datasets are available in section S1.1 of the Supplementary Materials.

The performance of GADMA2’s engines is evaluated on 2 simulated datasets: populations of fruit flies and orangutan species. For simulation purposes, we used a previously described scenarios available within the *stdpopsim* library [25]. Each dataset simulated by *msprime* engine [26] includes genetic data of 5 diploid individuals per each population.

**Figure 2:**
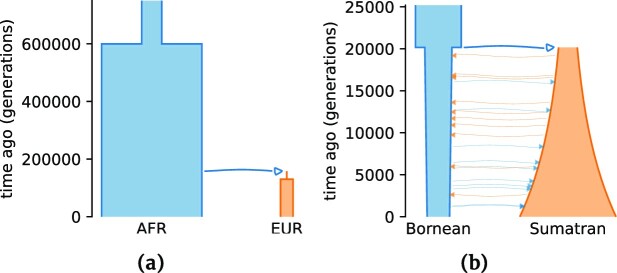
Demographic histories used in data simulations powered by *stdpopsim* [25] for performance comparison of GADMA2 likelihood engines. (a) History of African (AFR) and European (EUR) populations of *Drosophila melanogaster* from Li and Stephan [27]. (b) History of *Pongo pygmaeus* (Bornean) and *Pongo abelii* (Sumatran) orangutan species from Locke et al. [30].

Li and Stephan [27] presented the demographic history of *Drosophila melanogaster* populations from Africa and Europe. The visual representation of the history is shown in Fig. 2a. The African population is characterized by a single instantaneous expansion. The origin of the European population is a result of the divergence of a very limited number of individuals followed by instantaneous expansion. Five autosomal chromosomes with a total length of 0.11 Gbp are simulated under this demographic history. We use a mutation rate equal to 5.49 · 10^−9^ per base per generation [28] and a recombination rate of 8.4 · 10^−9^ per base per generation [29].

The demographic history of the Bornean (*Pongo pygmaeus*) and Sumatran (*Pongo abelii*) orangutans was originally inferred in Locke et al. [30] and is shown in Fig. 2B. Specifically, it is an isolation-with-migration history that describes the ancestral population split followed by the exponential growth of Sumatran and an exponential decline of Bornean orangutans. We simulate 23 autosomal chromosomes with a total length of 2.87 Gbp. The mutation rate used in the simulation is equal to 1.5 · 10^−8^ per site per generation [31]. Averaged recombination rates for each chromosome are taken from the *Pongo abelii* recombination map inferred in Nater et al. [31].

Datasets for the demographic inference of inbred species are taken from the original study by Blischak et al. [22]. The 11 × 5 AFS data for 2 populations of the American puma (*Puma concolor*) were constructed on the basis of Ochoa et al. [32]. The AFS data for 45 individuals of domesticated cabbage (*Brassica oleracea*) were obtained from publicly available resequencing data [33, 34]. Both allele frequency spectra are folded due to a lack of information about ancestral alleles. Datasets are presented in the repository of the original article and are available via the following link [35].

### Hyperparameter optimization

GADMA uses a genetic algorithm to optimize the demographic parameters [16]. A *hyperparameter* is usually defined as a parameter of an algorithm. The performance of any algorithm depends on its hyperparameters, and optimization of their values can significantly improve the overall efficiency. As an example of a hyperparameter, we can consider the number of demographic models in 1 iteration of the genetic algorithm. Several techniques can be used for the optimization of hyperparameters, and Bayesian optimization is one of the most popular methods [36]. The efficient method based on the Bayesian optimization is implemented in SMAC software [37, 38]. It has been applied in a number of studies, including optimization of neural networks [39–41].

SMAC addresses the algorithm configuration problem, which involves determining the optimal hyperparameters for a given algorithm across multiple instances. As such, it needs to solve a multiobjective optimization problem. To do this, SMAC uses a heuristic approach that optimizes a single objective function, the average value of the given cost function across the entire set of problem instances. Here we will refer to the objective function value as the *SMAC score*. The researcher usually selects the cost function according to the goal of the optimization process. This cost function is typically based on factors such as the time required to solve the problem or the quality of the solution achieved within a specific budget. It is important to exercise caution when working with SMAC score: since values of cost function are averaged across a given set of instances, it is essential that they have the same scale.

We use SMAC to tune the hyperparameters of the genetic algorithm in GADMA2. We divide all datasets into 2 groups of training and test datasets. The optimization is performed for GADMA’s genetic algorithm using *moments* engine and 4 training datasets as problem instances. Test datasets are used to validate the performance of the new configurations after optimization using SMAC. To ensure that the cost function has the same scale across all problem instances, we select the training datasets such that they have an identical number of populations and sample size. We choose the cost function as the best log-likelihood value achieved by GADMA’s genetic algorithm within a fixed number of likelihood evaluations. Regular GADMA’s pipeline, though, may require less or more evaluations to run depending on the dataset as it has a stop criterion that is based on the convergence. We take 200 times the number of dataset parameters as an allowed number of likelihood evaluations for the genetic algorithm runs in SMAC. Such a number of evaluations is a trade-off between speed and accuracy: according to the convergence plots, the convergence of default genetic algorithm optimization is slowing down at this point and is very close to the plateau walk (Supplementary Fig. S2, Supplementary Fig. S3). We note that we count the log-likelihood evaluations rather than the iterations of the genetic algorithm, as 1 iteration may involve multiple evaluations and the number of evaluations can vary across different configurations.

We perform several attempts of SMAC optimization for different variants of hyperparameter configurations. The descriptions and domains of all hyperparameters are given in Supplementary Table S1 and Table 1 correspondingly. Each attempt took 2 weeks in 10 parallel processes (Intel® Xeon® Gold 6248). First, optimization of all genetic algorithm hyperparameters is executed. Then, 2 discrete hyperparameters (gen_size and n_init_const) are fixed to 5 manually picked combinations of domain values. SMAC is used to find optimal continuous hyperparameters for each combination. Four combinations were excluded from the analysis. Hyperparameter gen_size that corresponds to the size of generation in a genetic algorithm is not tested for the value of 100 due to relatively slow convergence. This eliminates 3 combinations. Additionally, the constant of initial design n_init_const equal to 5 is excluded for a case of gen_size equal to 50 as it provides a small number of solutions for the first generation.

**Table 1:** Hyperparameters of the genetic algorithm in GADMA, their domains used in SMAC, and final values after each optimization attempt with SMAC. Hyperparameter values from attempt 1 are equal to the default GADMA values as SMAC failed to find a better configuration. For each of 2 to 6 attempts, 2 discrete hyperparameters (gen_size and n_init_const) are fixed in order to gain SMAC efficiency

		Attempt number					
Hyperparameter ID	Domain	1 (default)	2	3	4	5	6
gen_size	{10, 50, 100}	10	10*	10*	10*	50*	50*
n_init_const	{5, 10, 20}	10	10*	5*	20*	10*	20*
p_elitism	[0, 1]	0.20	0.30	0.30	0.30	0.40	0.40
p_mutation	[0, 1]	0.30	0.20	0.20	0.20	0.08	0.10
p_crossover	[0, 1]	0.30	0.30	0.30	0.30	0.42	0.46
p_random	[0, 1]	0.20	0.20	0.20	0.20	0.10	0.04
mutation_strength	[0, 1]	0.200	0.776	0.370	0.534	0.833	0.528
const_mutation_strength	[1, 2]	1.010	1.302	1.290	1.648	1.199	1.492
mutation_rate	[0, 1]	0.200	0.273	0.886	0.882	0.595	0.345
const_mutation_rate	[1, 2]	1.020	1.475	1.942	1.417	1.645	1.472

*These values are fixed during the hyperparameter optimization with SMAC.

Overall, we make 6 attempts of hyperparameter optimization using SMAC. Unlike the training datasets, the additional 6 test datasets are selected to be diverse and, as a result, have varying scales of their likelihood functions. Therefore, it is not correct to compare new configurations obtained from different SMAC attempts using the SMAC score calculated across both training and test datasets. Furthermore, the genetic algorithm within the SMAC framework was terminated earlier than during a regular GADMA run. Nevertheless, we first validate the efficiency of SMAC and make preliminary comparisons of the new configurations for the AFS-based engines (*moments*, ∂a∂i, and *momi2*) using SMAC scores evaluated across 128 independent runs. Then we compared new configurations by the log-likelihood values obtained from full genetic algorithm runs using the usual GADMA stop criterion. We use the same or equivalent criteria as the initial GADMA version to terminate the genetic algorithm [16]. For example, the genetic algorithm with a configuration with a generation size (gen_size) of 10 stops after 100 iterations without improvement, while the equivalent number of 20 iterations without improvement is used as a stop criterion for configurations with gen_size equal to 50.

The new configurations are compared as follows. First, we measure the speedup, which is the average fraction of the log-likelihood evaluations saved by a new configuration compared to the default configuration. Then, we compare each new configuration against the default configuration using the resulting likelihoods and determine its performance as *better, worse*, or *incomparable* for each dataset. For a fixed dataset, we consider a new configuration to be better if the median and both quartiles of the 128 likelihood values are higher than these quantities for the default configuration. If the median and both quartiles are lower, we consider the performance on the dataset to be worse. Otherwise, we declare the case incomparable. We aim to select a configuration taking into account both the speedup and the likelihood comparisons against the default configuration. Our goal is to find a configuration that is faster and performs better than the default configuration on as many datasets as possible, while also minimizing the number of datasets where it performs worse.

More information and details are available in section S1 of the Supplementary Materials.

### Performance test of GADMA2 engines

Four engines supported by GADMA2 (∂a∂i, *moments, momi2, momentsLD*) are compared on 2 simulated datasets of fruit fly populations and orangutan species. For each dataset, we test several models of the demographic history. The first 2 models are based on the ground-truth history used in the simulations but differ in the presence of migration. Then we infer parameters for 2 structure models with and without migration using the GADMA2 feature for automatic demographic inference. For the orangutan dataset, 3 additional models with pulse migrations are analyzed. The performance of all 4 engines is compared, but *momi2* engine is not tested for models with continuous migration as it does not support it. We run GADMA2 inference 8 times for each engine and model. Parameters of the history with the best log-likelihood are reported. Mutation and recombination rates for demographic inference are taken the same as in the data simulation. Their values are available in the Datasets section.

Using GADMA2 engines, we find and compare parameters for 4 models of *D. melanogaster* demographic history (Supplementary Table S5). Model DROS-NOMIG is an isolation model with instantaneous size change of the African population followed by separation of the European population, which experiences 2 epochs of constant sizes. Population sizes during these epochs are not dependent on each other. Model DROS-MIG describes the scenario identical to DROS-NOMIG but includes continuous asymmetric migration between populations from their divergence until presence. Both models DROS-NOMIG and DROS-MIG align with the original isolation history used for data simulation. Lastly, we test 2 models with (DROS-STRUCT-MIG) and without (DROS-STRUCT-NOMIG) migration for structure (2, 1). This notation means a model consisting of 2 epochs before the ancestral population split followed by divergence and 1 epoch for each of the 2 subpopulations. More details on model structure specification can be found in Noskova et al. [16]. By their definition, these structure models are misspecified due to simplification of the European population’s history: a 2-epoch scenario of the European population is approximated by 1 epoch with constant size, linear change, or exponential change.

We analyze engines’ performance on the orangutan dataset for 7 demographic models (Supplementary Table S13). Model ORAN-NOMIG is isolation with the ancestral population split followed by the exponential size changes of the Sumatran and Bornean orangutans. Model ORAN-MIG aligns with the history used in data simulation and describes an isolation-with-migration with the ancestral population split followed by the exponential size changes of the Sumatran and Bornean orangutans. An additional 2 models with structure (1, 1) without (ORAN-STRUCT-NOMIG) and with continuous migration (ORAN-STRUCT-MIG) are included in the analysis. We note that the original history contains gene flow and can be correctly estimated using ORAN-MIG and ORAN-STRUCT-MIG models.

In order to overcome *momi2*’s limitation on continuous migrations presented in the orangutan history, we tested the engine for additional demographic scenarios with pulse migrations. A different number of pulse migrations with equal rates are uniformly distributed within the epoch between the present time and species divergence time. ORAN-NOMIG and ORAN-STRUCT-NOMIG models are compared with 3 additional demographic models: (i) with 1 pulse migration (ORAN-PULSE1), (ii) with 3 pulse migrations (ORAN-PULSE3), and (iii) with 7 pulse migrations (ORAN-PULSE7).

### Inference of inbreeding coefficients

We perform demographic inference with GADMA2 using the data of the American pumas (*P. concolor*) and domesticated cabbage (*B. oleracea* var. *capitata*) from Blischak et al. [22]. For each dataset, parameters of 2 demographic models are inferred: (i) model from the original study without inbreeding and (ii) model from the original study with inbreeding. Each demographic inference is run 100 times, and the history with the highest log-likelihood value is selected. Two result histories are compared with the likelihood ratio test [42] to investigate which history best fits the data.

First, we use the same parameter bounds to repeat the demographic inference from Blischak et al. [22] with GADMA2. We compare the results of 100 runs of GADMA2 with the same number of results received using ∂a∂i’s optimization techniques. Then we perform another round of demographic inference with GADMA2 using wider bounds of parameters.

Performance of GADMA2 is compared with performance of 2 optimization techniques from ∂a∂i within the same setup as in Blischak et al. [22]. We first reproduce 100 launches of a single ∂a∂i’s optimization as they were conducted in the original study and measure the average number of evaluations and time of execution. Next, we run ∂a∂i’s optimization with restarts, meaning that the optimization is restarted multiple times for each run and the best log-likelihood parameters are considered as the result. In order to balance computational costs, number of restarts is determined to match the average number of evaluations of GADMA2. Notably, if average ∂a∂i’s single optimization run requires *X* likelihood evaluations and the average GADMA2 run involves *Y* evaluations, we compare the GADMA2 run with the run of ∂a∂i optimization with $\left\lceil {\frac{Y}{X}}\right\rceil$ restarts. We consider the number of evaluations for comparison of computational costs. It is a more reliable metric than the time of execution, as it is not affected by the specific hardware or parameter values used during optimization. Used optimization techniques from ∂a∂i require initial estimation of parameters, which can be done by sampling from a wide range of distributions. To ensure correct comparison, we use distribution from the GADMA2 initial design to perform this initialization.

We report and compare the mean, the standard deviation, and the best value of log-likelihood for 100 run repeats of GADMA2, of a single ∂a∂i’s optimization, and of the ∂a∂i’s optimization with restarts. The optimization methods used for ∂a∂i runs are the BFGS algorithm [43–46] for the American puma data and the BOBYQA method [47] for the domesticated cabbage data, as described in Blischak et al. [22].

Mutation rates, generation times, and sequence lengths for parameter translation were taken from Blischak et al. [22]. Demographic parameters for *P. concolor* are translated from the genetic to real units using a mutation rate of μ = 2.2 × 10^−9^, a generation time of 3 years, and a sequence length of 2,564,692,624 bp [32]. In the case of *B. oleracea* var. *capitata*, population demographic parameters are translated using a mutation rate of μ = 1.5 × 10^−8^, a generation time of 1 year, and a sequence length of 411,560,319 bp.

The Godambe information matrix approach [42] was used for evaluation of the confidence intervals (CIs) in the original study [22]. This approach requires step size ϵ to estimate parameters’ uncertainty. The value of step size can influence the stability of Godambe approximation, and several values should be tested to confirm consistent results between them. As in Blischak et al. [22,] we estimate and compare uncertainties across a range of step sizes: 10^−2^ − 10^−7^ by factors of 10. Reported confidence intervals for the final histories are estimated on 100 bootstrapped AFS data from the original study using the Godambe information matrix with a step size equal to ϵ = 10^−2^ [42]. The scripts and data used for CI evaluation are taken from the repository [35] of Blischak et al. [22].

## Results and Discussion

### Updated genetic algorithm

The genetic algorithm in GADMA2 is improved by the hyperparameter optimization implemented in SMAC software [37, 38]. Ten hyperparameters (Table 1) of the genetic algorithm were optimized during the first optimization attempt. SMAC performed 13,900 runs of the genetic algorithm and tested 2,222 different hyperparameter configurations. This process took 2 weeks of continuous computations on cluster. However, SMAC failed to find a better solution than the default one. We assume that such behavior may be caused by the presence of 2 discrete hyperparameters in the configuration. These hyperparameters are fixed to 5 specific combinations of the domain values during the next attempts of SMAC-based optimization of the remaining continuous hyperparameters.

As a result, we perform 6 attempts of hyperparameter optimization for different configurations of GADMA2, and the result configurations are presented in Table 1. For each of these new configurations, we manually evaluate the SMAC scores using 128 independent runs for each dataset and engine (*moments*, ∂a∂i, and *momi2*). They can be found in Supplementary Table S2 for the *moments* engine, Supplementary Table S3 for the ∂a∂i engine, and Supplementary Table S4 for the *momi2* engine. The costs and results for ∂a∂i are very similar to those for *moments*, supporting the idea that ∂a∂i and *moments* engines have very similar performance. Based on the obtained SMAC scores, the attempt 3 configuration is the best for *moments* and *momi2* engines and second best for the ∂a∂i engine. However, we do not rely solely on the mean SMAC score as a selection criterion for these new configurations. This is because during SMAC runs, the genetic algorithm was stopped earlier, and its full run performance may be different. Additionally, log-likelihoods between test datasets and between engines have different scales, making direct comparison difficult.

To address this problem, we determine the best new configuration based on the performance of 128 full genetic algorithm runs using *moments* and *momi2* engines. For each configuration, we measure the average speedup and indicate whether or not the result likelihood is better than for the default configuration. We do not perform the full runs for the ∂a∂i engine due to high computational costs and its similarity to the *moments* engine. The boxplots of log-likelihood values and the required number of evaluations are presented in Supplementary Fig. S4 for the *moments* engine and Supplementary Fig. S10 for the *momi2* engine. The convergence plots of a genetic algorithm with different configurations on training and test datasets are presented in Supplementary Figs. S2 and S3 for the*moments* engine, Supplementary Figs. S6 and S7 for the ∂a∂i engine, and Supplementary Figs. S8 and S9 for the *momi2* engine. The dataset counts for which new configurations demonstrate better, worse, and incomparable performance comparing to the default configuration are presented in Supplementary Fig. S5 for the *moments* engine and in Supplementary Fig. S11 for the *momi2* engine.

**Figure 3:**
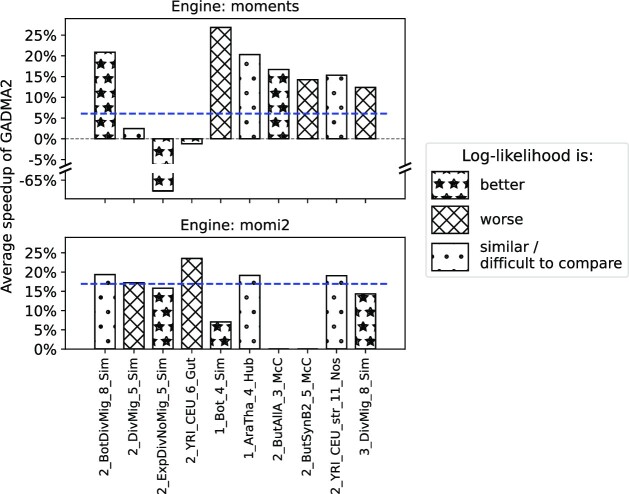
Performance comparison of the initial GADMA and GADMA2 with new hyperparameters. Bar size illustrates the average speedup of GADMA2, defined as the fraction of log-likelihood evaluations saved by the new version for each dataset. Blue dashed line demonstrates the average fraction of saved evaluations across all datasets. The bars’ hatching patterns indicate the improvement of the result log-likelihood based on median and quartiles. GADMA2 with new hyperparameters attains the average speedup of 10% and provides better results on average compared to the default configuration.

**Figure 4:**
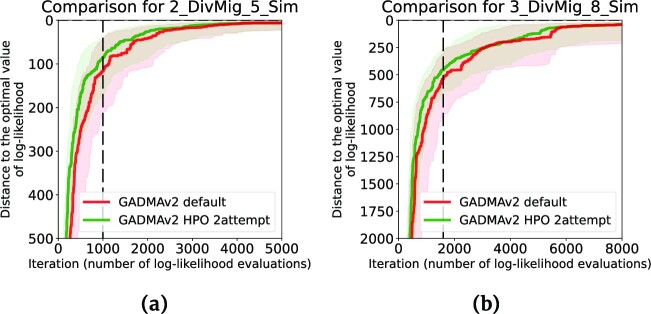
Example convergence plots for the default genetic algorithm configuration from the initial version of GADMA (red) and configuration obtained during attempt 2 of hyperparameter optimization with SMAC (green) on 2 datasets: (a) training dataset 2_DivMig_5_Sim and (b) test dataset 3_DivMig_8_Sim. For each configuration, 128 independent optimization runs were performed. Solid lines correspond to median convergence over 128 run,s and shadowed areas are ranges between the first (0.25) and third (0.75) quartiles. The vertical dashed black line refers to the number of evaluations used to stop a genetic algorithm in SMAC.

On most datasets, all new configurations require a smaller number of evaluations than the default genetic algorithm (Supplementary Figs. S4 and S10). There is only 1 dataset, 2_ExpNoMig_5_Sim (*moments* engine), for which the default configuration performs faster than all new configurations. In general, the configurations from attempts 3, 5, and 6 are the fastest. However, their log-likelihoods are worse than the default configuration on most datasets (Supplementary Figs. S5 and S11). Configurations from attempts 2 and 4 demonstrate best performance in terms of resulting likelihoods among new configurations. Moreover, the genetic algorithm with hyperparameters from attempt 2 has better log-likelihood results for the *moments* engine while the configuration from attempt 4 has better performance for the *momi2* engine. Since the hyperparameter optimization used the *moments* engine, we choose the configuration from attempt 2 for the genetic algorithm in GADMA2. Figure 3 summarizes the improvement obtained by GADMA2 with the new hyperparameters as compared to the initial version. The new configuration saves around 10% of evaluations and provides better results on average compared to the default genetic algorithm. Some examples of the convergence plots that compare the previous version of the genetic algorithm and the new version of genetic algorithm are presented in Fig. 4.

### Flexible structure model

Automatic demographic model construction is a central feature of GADMA. It replaces the fully manual choice of a model with a *model structure* specification. Traditionally, demographic models only have continuous parameters. Demographic structures, on the other hand, define the number of epochs before, after, and between population splitting events and assign a discrete variable representing population dynamics type to each epoch. GADMA optimizes over these discrete variables alongside with the usual continuous ones, examining what would be a multitude of models in the traditional sense. GADMA2 gives the user more control over the search space in this setting.

#### Migration rates

One of the existing controls over model parameters is the opportunity to disable all migration events and to infer demographic history without any gene flow. GADMA2 now includes a new control handle to make migrations symmetric. Additionally, it allows for specific migrations to be disabled by setting up migration masks.

#### Selection and dominance rates

Both of the initially supported likelihood engines included in GADMA, ∂a∂i, and *moments* are able to infer selection and dominance rates. This inference approach, first presented in Williamson et al. [48], assumes a single selection rate for the entire population while real genetic data could consist of regions with different rates. Despite this simplification, such inference can provide useful estimations of selection. The first version of GADMA lacked the function to make these inferences, and we have added these in the new version. GADMA2 enables the approximation of selection rates and dominance coefficients for automatically constructed demographic models.

#### Population size dynamics

GADMA2 provides additional flexibility for population size estimation during model construction. Previously, demographic parameters such as functions of population size changes were estimated within a fixed set of 3 possible dynamics: constant, linear, or exponential change. Now, the list of available population size dynamics in GADMA2 can be appointed to any subset of 3 basic functions. Thus, for example, linear size change can be excluded from the demographic inference if only constant and exponential dynamics are applicable, like in the case of the *momi2* engine.

#### Inbreeding coefficients

Since the publication of the first version of GADMA, the supported likelihood engines were also upgraded. GADMA2 follows these changes and includes inference of inbreeding coefficients that were implemented in ∂a∂i [22]. Using this new feature included in ∂a∂i, we demonstrate that GADMA2 provides better and more stable results for inference of the demographic models obtained from data for the puma and cabbage reported by Blischak et al. [22] (Supplementary Tables S21, S22, S26, and S27).

### Data formats

Another improvement of ∂a∂i and *moments* is the ability to build an AFS dataset directly from a VCF file. Before this feature was implemented, this had to be done either manually or using another software like *easySFS* [49]. GADMA2 is able to read data directly from a VCF file and downsize, exclude populations from, or build a folded AFS automatically. Such a feature allows broader and more convenient usage of GADMA2.

### New likelihood engines

In addition to ∂a∂i and *moments*, GADMA2 now includes 2 new likelihood engines: *momi2* [10] and *momentsLD* [14, 15]. Thus, 4 engines are provided in the common interface of GADMA2. Both ∂a∂i and *moments* engines are based on the Wright–Fisher diffusion and use allele frequency spectrum statistics for demographic inference.

*Momi2* implements a structured coalescent–backward-in-time stochastic process that is dual to the Wright–Fisher diffusion yet scales well to a large number of populations. It also uses AFS data as ∂a∂i and *moments* but is computationally faster and can handle up to 10 populations. However, *momi2* does not support continuous migration and linear change of population size.

Even though the allele frequency spectrum is one of the most popular statistics for demographic inference, it has limitations on how informative it can be [50]. The software *moments* has a submodule *momentsLD* dedicated to demographic inference using LD statistics. In general, low-order 2-locus LD statistics are used in *momentsLD*. A new likelihood engine using *momentsLD* is the first engine in GADMA that does not use AFS-based statistics.

Overall, GADMA2 now provides a choice of 4 likelihood engines, and we encourage the community to extend this list.

### A new engine for demographic history representation

During demographic inference, GADMA provides different textual and visual representations of the current best demographic history, such as generated Python code for all available likelihood engines or picture with visualized demographic history. Recently, a new Python package named *demes* [51] appeared to allow standard human-readable descriptions of demographic histories. GADMA2 includes *demes* as an engine to generate native descriptions and plots of demographic histories, which was only possible before using the *moments* or *momi2* engine. Figure 2 shows the examples of visual representations of demographic history using *demes*.

### Performance comparison of GADMA2 engines

We compare 4 likelihood engines supported by GADMA2 on 2 simulated datasets of fruit flies and orangutans. Several demographic models are used. Their description is provided in the Performance test of GADMA2 engines section of Materials and Methods.

The simulated parameter values of *D. melanogaster* population history and their estimations inferred by engines in GADMA2 are presented in Table 2 and Supplementary Tables S7, S8, and S9 for DROS-NOMIG, DROS-MIG, DROS-STRUCT-NOMIG. and DROS-STRUCT-MIG models correspondingly. The mean time of 1 log-likelihood evaluation and the mean number of evaluations averaged over inference runs are reported in Supplementary Tables S9 and S10.

**Table 2:** The demographic parameters of *Drosophila melanogaster* history without migration (DROS-NOMIG model) inferred with different engines in GADMA2. Ground truth includes the parameter values from Li and Stephan [27] used in simulation powered by *stdpopsim* [25]. Log-likelihood values are not comparable between different engines

		∂a∂i	*moments*	*momi2*	*momentsLD*
Log-likelihood	Ground truth	−2,808	−1,101	−53,489,812	−268
Parameter					
*N_anc_*	1,720,600	1,598,851	1,580,074	1,724,622	1,243,096
*N_AFR_*	8,603,000	8,421,135	7,949,903	8,679,000	7,963,770
*N*_*EUP*0_	2,200	21,999	16,158	439	2,013
*N_EUP_*	1,075,000	1,082,115	1,008,276	1,008,970	1,102,340
*T_AFR_* (gen.)	600,000	603,933	560,015	597,487	715,948
*T_split_* (gen.)	158,000	173,658	162,631	159,205	153,248
*T_EUP_* (gen.)	154,600	137,587	136,450	158,503	149,942

*N_anc_*, size of the ancestral population; *N_AFR_*, size of the African population after expansion; *N*_*EUP*0_, European bottleneck population size after divergence; *N_EUP_*, modern size of the European population; *T_AFR_*, time of African size expansion; *T_split_*, time of divergence; *T_EUP_*, time of European expansion.

Estimations of orangutan history model parameters and their ground-truth values are available in Supplementary Tables S14, S15, and S16 and Table 3 for ORAN-NOMIG, ORAN-MIG, ORAN-STRUCT-NOMIG, and ORAN-STRUCT-MIG models, respectively. The results of parameter estimations using *momi2* for models with 0, 1, 3, and 7 pulse migrations are presented in Table 4. The average time of 1 log-likelihood evaluation and the mean number of evaluations for used models and engines are reported in Supplementary Tables S19 and S20.

**Table 3:** The demographic parameters of orangutan history with migration for structure (1, 1) (ORAN-STRUCT-MIG model) inferred with different engines in GADMA2. Ground truth includes the simulated parameter values that were obtained from the original study by Locke et al. [30]. *Momi2* engine was excluded as it does not support continuous migrations. Log-likelihood values are not comparable between different engines

		∂a∂i	*moments*	*momentsLD*
Log-likelihood	Ground truth	−1,220	−1,106	−53
Parameters				
*N_anc_*	17,934	17,925	17,854	17,685
$N_{Bor\_split}$	10,617	10,432	10,498	10,529
$N_{Sum\_split}$	7,317	7,492	7,355	7,155
*N_Bor_*	8,805^*exp*^	9,282^*exp*^	8,892^*exp*^	8,592^*exp*^
*N_Sum_*	37,661^*exp*^	39,343^*exp*^	37,443^*exp*^	36,740^*exp*^
*m*_*Bor* − *Sum*_( × 10^−5^)	0.66	0.67	0.67	0.69
*m*_*Sum* − *Bor*_( × 10^−5^)	1.10	1.07	1.09	1.13
*T_split_* (gen.)	20,157	20,812	20,183	19,869

*N_anc_*, size of the ancestral population; $N_{Bor\_split}$, size of *Pongo pygmaeus* at split; $N_{Sum\_split}$, size of *Pongo abelii* at split; *N_Bor_*, modern size of *Pongo pygmaeus; N_Sum_*, modern size of *Pongo abelii; m*_*Bor* − *Sum*_, migration rate from *Pongo pygmaeus* to *Pongo abelii; m*_*Sum* − *Bor*_, migration rate from *Pongo abelii* to *Pongo pygmaeus; T_split_*, time of divergence.

^*exp*^Exponential growth.

**Table 4:** The demographic parameters of orangutan histories without migration and with pulse migrations inferred using the *momi2* engine in GADMA2. In ORAN-PULSE* models, the time interval after divergence is divided into equal parts, and pulse migrations are integrated between them. The inferred parameters show convergence to true values with an increase in pulse migration number. Ground truth includes the simulated parameter values obtained from the original study by Locke et al. [30]

		Model ORAN-				
	Ground truth	NOMIG	STRUCT-NOMIG	PULSE1	PULSE3	PULSE7
Number of pulse migrations	0 (continuous)	0	0	1	3	7
Log-likelihood		−48,541,453	−48,545,934	−48,437,315	−48,391,684	−48,377,617
Parameters						
*N_anc_*	17,934	19,331	19,086	19,220	18,461	17,997
$N_{Bor\_split}$	10,617	6,187	8,453	8,731	8,715	10,086
$N_{Sum\_split}$	7,317	7,719	11,668	4,165	5,412	6,409
*N_Bor_*	8,805	10,663	8,453	9,631	9,640	8,768
*N_Sum_*	37,661	54,184	49,595	59,929	43,123	38,030
*m*_*Bor* − *Sum*_	0.66 × 10^−5^	0	0	0.065	0.057	0.025
*m*_*Sum* − *Bor*_	1.10 × 10^−5^	0	0	0.206	0.084	0.036
*T_split_* (gen.)	20,157	11,270	11,668	16,211	20,086	20,809

*N_anc_*, size of ancestral population; $N_{Bor\_split}$, size of *Pongo pygmaeus* at split; $N_{Sum\_split}$, size of *Pongo abelii* at split; *N_Bor_*, size of *Pongo pygmaeus* after exponential decline; *N_Sum_*, size of *Pongo abelii* after exponential size change; *m*_*Bor* − *Sum*_, migration rate from *Pongo pygmaeus* to *Pongo abelii; m*_*Sum* − *Bor*_, migration rate from *Pongo abelii* to *Pongo pygmaeus; T_split_*, time of divergence in generations.

Below we present our general conclusions about the results. A more detailed comparison is available in section S2 of the Supplementary Materials.

#### Fruit fly demographic history

Parameter values for models DROS-NOMIG and DROS-MIG that align with the ground truth are inferred accurately by all tested likelihood engines. Best estimations are obtained for the DROS-NOMIG model using the *momi2* engine. The bottleneck European population size is approximated most accurately by the *momentsLD* engine. Result histories for model DROS-MIG have worse values of log-likelihood than histories for the DROS-NOMIG model. Since DROS-NOMIG and DROS-MIG models are nested, this indicates optimization failure. Nevertheless, they are able to catch general history and low migration rates. Thus, based on these results, it is possible to assume population isolation and use further models without migrations for more accurate estimations.

We observe interesting results for the misspecified models with structure (2, 1). In the case of the DROS-STRUCT-NOMIG model, the ground-truth history of *D. melanogaster* is accurately approximated by *moments* and *momentsLD* engines only. The 2-epoch history of the European population is approximated by exponential growth with a rate that differs between engines (Supplementary Fig. S12). The approximation made using the *moments* engine aligns more closely with the actual history in terms of the mean population size and coalescent time, while the approximation from *momentsLD* is more accurate in terms of harmonic mean population size (section S2.1.1 of the Supplementary Materials). We note that the *momentsLD* engine also is able to provide similar history for the model DROS-STRUCT-MIG with migrations. However, ∂a∂i, *momi2*, and *moments* for both models are hindered by the severe local optimum and were not able to achieve a global solution within 8 GADMA2 runs. The alternative history is able to catch the European population history and low migration rates, yet it does not reflect the instantaneous expansion of the ancestral population, and the parameter value for the African population size hits the upper bound. Using models with African population size fixed to the ancestral population size after expansion helps to overcome the local optimum and achieve history similar to the ground truth (section S2.1.2 of the Supplementary Materials, Supplementary Tables S11 and S12).

#### Orangutan demographic history

In the case of the orangutan simulated dataset, all 4 engines provide similar demographic histories for the ORAN-NOMIG model without migrations. The predicted parameters are almost identical for ∂a∂i, *moments*, and *momi2*, which are AFS-based engines. Estimations for the modern sizes of populations are greater than the actual values used for the simulation. Moreover, the time of divergence is estimated to be lower: ∼12,000 vs. ground truth of ∼20,000. These discrepancies between predicted and simulated parameter values for the model ORAN-NOMIG could be explained by the fact that the model is oversimplified and lacks migration.

Model ORAN-MIG aligns correctly with the history used for data simulation. All tested engines provide estimations close to the simulated parameter values for the ORAN-MIG model.

The result demographic parameters for the ORAN-STRUCT-NOMIG model are close to the estimations obtained for the ORAN-NOMIG model. The population size dynamics are correctly inferred to be exponential for ∂a∂i and *momentsLD* engines. However, *momi2* and *moments* predict the constant size of the Bornean population. Although constant size approximates the Bornean population history relatively well, we demonstrate that our result is a consequence of the following model restriction. The model ORAN-STRUCT-NOMIG obliges the sum of Sumatran and Bornean population sizes after divergence to equal the ancestral population size. Ground-truth history follows this rule, but it is not fulfilled by the estimations inferred for the ORAN-NOMIG model. We additionally test the ORAN-NOMIG model with the same restriction on population sizes for *momi2* and *moments* engines. The best obtained scenarios have a worse log-likelihood value than histories with constant size of the Bornean population obtained for the ORAN-STRUCT-NOMIG model (section S2.2 of the Supplementary Materials).

Moreover, we remove the restriction on population sizes for the ORAN-STRUCT-NOMIG model and infer parameters for the new modified model using *moments* and *momi2* engines. The history received for *momi2* engine is similar to those obtained for the ORAN-NOMIG model, and the history of Bornean population is estimated correctly by the exponential dynamic. However, even though the *moments* engine also assumes exponential size change for the Bornean population, it approximates the exponential growth of Sumatran population size by a linear dynamic. Yet the history with linear approximation is similar to other histories obtained by *moments* for models ORAN-NOMIG and ORAN-STRUCT-NOMIG without migration. Furthermore, we ensure that such a model without the restriction but with linear size change is considered better than the result history for the ORAN-NOMIG model not only by the *moments* engine but also by ∂a∂i and *momentsLD* (section S2.2 of the Supplementary Materials). Thus, we have observed that model misspecifications like absence of migrations may lead to confusion between exponential and linear dynamics, but the results will still reflect the ground-truth history.

The original demographic history of orangutan species used for data simulation is accurately reconstructed by ∂a∂i, *moments*, and *momentsLD* engines within the ORAN-STRUCT-MIG model. Population size dynamics are inferred to be exponential for all tested engines. The parameters and values of log-likelihood are similar to the results for the ORAN-MIG model.

Finally, we analyze *momi2* engine performance for additional model ORAN-PULSE* with pulse events (Table 4). Pulse migration rates inferred by *momi2* differ significantly from continuous rates used in the simulation. However, it is important to note that pulse migration rates cannot be directly compared to continuous migration rates. As the number of pulse events increases, we expect the rates to decrease, and it is supported by our results. For example, the migration rate from Bornean orangutans to Sumatran orangutans (*m*_*Bor* − *Sum*_) is inferred to be equal to 0.65 for model ORAN-PULSE1 with 1 pulse migration, 0.057 for model ORAN-PULSE3 with 3 pulses, and 0.025 for model ORAN-PULSE7 with 7 pulse events. It is crucial that other parameters converge to the simulated parameter values with an increased number of pulse events. Along these lines, population divergence time is estimated to be ∼11,000 generations for models ORAN-NOMIG and ORAN-STRUCT-NOMIG, ∼16,000 generations for the model ORAN-PULSE1, and ∼20,000 for models ORAN-PULSE3 and ORAN-PULSE7. The latter is close to the value of 20,157 used in the simulation. Parameter estimations for model ORAN-PULSE7 with 7 pulse migrations are the most accurate among tested models. We assume the increase in pulse events number will lead to more accurate estimations yet require more computational resources. Thus, continuous migration is not supported in the *momi2* engine but, to some degree, could be replaced by several pulse migration events.

### Usage case: inference of inbreeding coefficients

We use GADMA2 to reproduce demographic inference from Blischak et al. [22] for datasets of American pumas (*P. concolor*) and domesticated cabbage (*B. oleracea* var. *capitata*). Blischak et al. [22] performed the demographic inference for 2 models without (model 1) and with inbreeding (model 2) using ∂a∂i’s optimization approaches.

First, we run GADMA2 with the ∂a∂i engine and the same parameter bounds as in Blischak et al. [22] and compare the results of 100 repeats with the results obtained by ∂a∂i’s optimization techniques. The result statistics, such as mean number of evaluations, mean execution time, and mean and best values of log-likelihood, are presented in Supplementary Tables S21 and S22 for American pumas and Supplementary Tables S26 and S27 for domesticated cabbage. They demonstrate that on average, a single GADMA2 run provides better and more stable results than a single run of ∂a∂i’s optimization within 100 repeats. However, when optimization from ∂a∂i is restarted several times in order to match the computational costs of GADMA2, the results are not so consistent. In case of American puma populations, final average and best log-likelihood values for GADMA2 are better than for ∂a∂i’s optimization with restarts. For domesticated cabbage inference, optimization from ∂a∂i with restarts attains better average results than GADMA2. Yet number of restarts required to cover GADMA2 computational costs differs a lot between datasets and models and is always unknown in practice.

**Figure 5:**
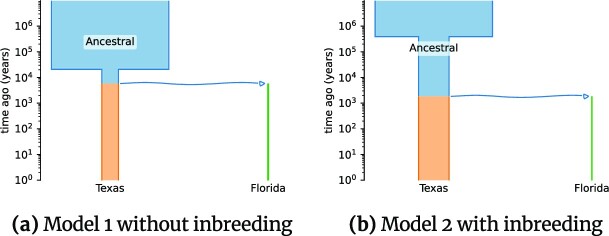
Demographic histories for Texas and Florida populations of American puma inferred with GADMA2. Figures are generated with the *demes* package [51]. Time is presented on a log scale.

**Figure 6:**
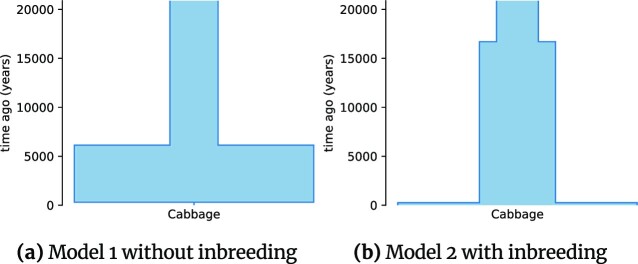
Demographic histories for a single population of domesticated cabbage inferred with GADMA2. Figures are generated with the *demes* package. In both models, the time of the most recent epoch is estimated to be small.

Several parameters of the result demographic histories obtained during first GADMA2 inference for both datasets received values close to their upper or lower bounds. In order to overcome this limitation, we perform another inference with wider bounds for parameter values and observe more reliable demographic parameters. The final values of the parameters and their CIs are presented in Supplementary Table S25 for American pumas and Supplementary Table S30 for domesticated cabbage. Uncertainty estimates for CI evaluation are consistent across different step sizes and are presented in Supplementary Tables S23 and S24 for American pumas and in Supplementary Tables S28 and S29 for domesticated cabbage. The visual representations of demographic histories using *demes* can be found in Fig. 5 for American pumas and in Fig. 6 for domesticated cabbage.

#### American puma demographic history

The best demographic histories obtained with GADMA2 have better values of log-likelihood (−452,492.70 vs. −453,003.05 for model 1 and −316,115.56 vs. −318,058.08 for model 2) than those reported in Blischak et al. [22]. Similar values of population sizes are obtained except for the size of the Florida population, which is estimated to be 860 and 374 individuals for model 1 and model 2, respectively, compared to the 1,200 and 1,600 individuals estimated by Blischak et al. [22]. Time of divergence is estimated as 5,800 years ago for model 1 and as 1,800 for model 2. Inbreeding coefficients for model 2 are reported to be slightly higher than for the same model in Blischak et al. [22]: 0.453 for the Texas population and 0.628 for the Florida population. The Godambe-adjusted likelihood ratio test (LRT) statistic is 2,634.18 (*P* = ∼0.0; Coffman et al. [42]), indicating that the model with inbreeding better describes data.

#### Domesticated cabbage demographic history

The best demographic histories obtained with GADMA2 for the domesticated cabbage population have better log-likelihood values (−24,137.34 vs. −24,330.40 for model 1 and −4,267.32 vs. −4,281.14 for model 2) than those received in Blischak et al. [22]. Values for the population sizes in the first and second epochs are inferred similar to the results from Blischak et al. [22]. However, the population size estimation for the most recent epoch in our results is lower (10 vs. 592 individuals) for model 1 without inbreeding and higher (174,960,000 vs. 215,000 individuals) for model 2 with inbreeding than estimates obtained by ∂a∂i in [22]. The time duration of the epoch is also smaller for both models than estimated previously. In the case of model 1, the time parameter is very close to zero. The likelihood ratio test showed that the model with inbreeding better describes the data than the model without inbreeding (LRT statistic = 126.59, *P* = ∼0.0; Coffman et al. [42]).

## Conclusions

GADMA2 is an extension of GADMA. It features an improved genetic algorithm, a more flexible automatic model construction setup, and 2 additional demographic likelihood engines. We showcased GADMA2 by comparing different likelihood engines for 2 simulated datasets with various demographic models, including misspecified ones. Furthermore, we applied GADMA2 to infer demographic histories for 2 empirical datasets of inbred species, reporting updated parameters.

To improve the genetic algorithm, we ran hyperparameter optimization powered by SMAC. We observed that discrete hyperparameters might hinder hyperparameter optimization, requiring much more iterations. Because of this, we manually picked 5 combinations of the discrete hyperparameters, running SMAC-based optimization of the remaining continuous ones for each fixed combination. We compared the set of optimal solutions on various datasets for 3 AFS-based likelihood engines of GADMA2: ∂a∂i, *moments*, and *momi2*. We included the configuration that performed best when averaged across all likelihood engines as GADMA2’s new genetic algorithm. It is worth noting, however, that there was no one configuration that performed best for all of the likelihood engines simultaneously. We thus propose introducing engine-specific genetic algorithm configurations as a valuable direction for future work. Another prospective direction is population count-specific configurations (i.e., making the optimization of GADMA different for 1, 2, and 3 populations). An important point here is that log-likelihoods for different datasets with a fixed population count are more comparable to each other. This could help SMAC’s heuristic target function better reflect the real multiobjective goal and thus improve its hyperparameter optimization performance.

GADMA’s automatic model construction setup was improved to allow forbidding specific migrations or making them symmetric, as well as restricting the admissible types of population size dynamics. Moreover, inference of selection and inbreeding coefficients was made possible in GADMA2. We note that the approach included in GADMA2 for inference of the selection and dominance rates is limited and can provide only simplified estimations. In order to perform more accurate inference of selection, other approaches should be used [52].

Two new demographic likelihood engines, *momi2* and *momentsLD*, were incorporated into GADMA2. The former is based on a different mathematical model than ∂a∂i and *moments* and is computationally faster than them, but it does not support continuous migrations and linear population size growth. The latter is the first engine in GADMA2 that does not use allele frequency spectrum data for demographic inference and relies on linkage disequilibrium statistics instead. Furthermore, the new package *demes* was incorporated into GADMA2 as a representation engine providing textual and visual descriptions of demographic histories.

We analyzed the accuracy of GADMA2’s demographic likelihood engines on 2 simulated datasets: the dataset of fruit fly populations and the dataset of orangutan species. We used different demographic models, including models with structures. Some of these models align with the ground truth, while some are misspecified due to various simplifications. Similar performance was observed over all engines for the models that align with the ground truth. In this case, inferred demographic histories were close to the ground truth, and the types of population size dynamics were correctly recovered for models with structures.

Demographic inference with the misspecified models demonstrated interesting phenomena. For the misspecified models with structure and the fruit fly dataset, all the AFS-based engines were stuck at the same local optimum. However, the resulting demographic histories were still able to give some insights about the studied populations. The new LD-based engine *momentsLD* performed considerably better than the AFS-based engines. For the orangutan dataset, both the AFS-based engines and *momentsLD* performed well. All slight discrepancies between estimated and ground-truth values are consequences of models’ restrictions and misspecifications. It was found that sometimes exponential growth of population size could be misconstrued as linear growth with a similar rate for misspecified models that do not include migration events. Although the *momi2* engine does not support continuous migrations required to accurately model the ground truth, it performs well in approximating these with a number of pulse migrations. However, this approach is limited because larger numbers of pulse migrations increase computation time.

GADMA2 greatly simplifies performing such comparisons, the in-depth study of which seems a prospective work direction.

We reproduced the demographic inference setup of Blischak et al. [22] for the datasets of American pumas and domesticated cabbage. We compared performance of the fully ∂a∂i-based inference to GADMA2. GADMA2 attained higher log-likelihoods with lower variance across different runs than a single ∂a∂i optimization. GADMA2’s run times, however, are considerably longer than ∂a∂i’s, to the extent that ∂a∂i’s optimization can sometimes yield better results when restarted multiple times to match the computational costs of GADMA2. However, it may be difficult to determine the number of restarts needed, while GADMA2’s automatic termination makes for a simpler and, arguably, more reliable user experience.

Finally, we found updated parameters for models, both with and without inbreeding, for the datasets of American pumas and domesticated cabbage from Blischak et al. [22]. For each dataset, the best demographic histories include inbreeding. Our results, however, demonstrate very broad CIs for some model parameters. The wide CIs for the population size of domesticated cabbage during the most recent epoch can be explained by the fact that epoch length was inferred to be small, and very recent events are difficult to investigate with the ∂a∂i engine. However, the same results for the size of the Florida puma population and the population divergence time are difficult to explain. We only tested the demographic models from Blischak et al. [22]; new models, however, can be built based on our results.

GADMA2 extends the GADMA that has already shown itself as powerful and efficient software for the inference of complex demographic histories from genetic data. With its new application programming interface, GADMA2 can be easily improved further by integrating new likelihood engines, new optimization algorithms, and automatic model construction routines.

## Availability of Source Code and Requirements

GADMA2 is freely available from GitHub via the link https://github.com/ctlab/GADMA and can be easily installed via Pip or BioConda. Detailed documentation is located on the website [53] and includes a user manual, ready-to-use examples, and a section about the Application Programming Interface (API). API enables an opportunity to use GADMA2 as a Python package and allows its optimization algorithms to be applied to any general optimization problem. An example of such usage is demonstrated for Rosenbrook function [54] optimization and is provided in the documentation.

- Project name: GADMA
- Version: 2.0.0
- Project homepage: https://github.com/ctlab/GADMA
- Documentation: https://gadma.readthedocs.io
- RRID: RRID:SCR_017680
- biotoolsID: biotools:GADMA
- Operating system(s): Platform independent
- Programming language: Python
- Other requirements: Python3.6 or higher, other requirements are available within the documentation
- License: GNU GPL v3

## Supplementary Material

giad059_GIGA-D-22-00279_Original_Submission

giad059_GIGA-D-22-00279_Revision_1

giad059_GIGA-D-22-00279_Revision_2

giad059_GIGA-D-22-00279_Revision_3

giad059_Response_to_Reviewer_Comments_Original_Submission

giad059_Response_to_Reviewer_Comments_Revision_1

giad059_Response_to_Reviewer_Comments_Revision_2

giad059_Reviewer_1_Report_Original_Submission Ryan Gutenkunst -- 11/7/2022 Reviewed

giad059_Reviewer_1_Report_Revision_1 Ryan Gutenkunst -- 2/10/2023 Reviewed

giad059_Reviewer_2_Report_Original_Submission Ilan Gronau -- 12/2/2022 Reviewed

giad059_Reviewer_2_Report_Revision_1 Ilan Gronau -- 2/14/2023 Reviewed

giad059_Supplemental_File

## Data Availability

An archival copy of the code and other supporting data, also including scripts to reproduce the figures, are available via the *GigaScience* repository, GigaDB [55]. The scripts and the results of hyperparameter optimization experiments are saved in the repository and available via the link [56]. The results of GADMA runs for different hyperparameter configurations are stored as an archive available in the *GigaScience* repository, GigaDB [55]. The results of experiments about inbreeding are added to the repository with the final demographic histories inferred in the original paper of GADMA and are located via the link [57].

## Additional Files

**Supplementary Fig. S1**. The model of the demographic history and naming convention of the example dataset from the deminf data v1.0.0 package.

**Supplementary Fig. S2**. Convergence plots for 6 genetic algorithm configurations using the *moments* engine on 4 training datasets: (1) the default genetic algorithm from the initial version of GADMA and (2–6) configurations obtained during attempts 2 to 6 of hyperparameter optimization with SMAC. The abscissa presents the log-likelihood evaluation number; the ordinate refers to the distance to the optimal value of log-likelihood. Solid lines correspond to median convergence over 128 runs and shadowed areas are ranges between first (0.25) and third (0.75) quartiles. The vertical dashed black line refers to the number of evaluations used to stop a genetic algorithm in SMAC. The default configuration (red) and 2 configurations from attempt 2 (green) and attempt 6 (blue) were compared in terms of convergence on a greater number of iterations. The configuration from attempt 2 shows faster convergence on first iterations, and the configuration from attempt 6 turns out to have better convergence at the last iterations on 3 of 4 datasets.

**Supplementary Fig. S3**. Convergence plots for 6 genetic algorithm configurations using the *moments* engine on 6 test datasets: (1) the default genetic algorithm from the initial version of GADMA and (2–6) configurations obtained during attempts 2 to 6 of hyperparameter optimization with SMAC. The default configuration (red) and 2 configurations from attempt 2 (green) and attempt 6 (blue) were compared in terms of convergence on a greater number of iterations. The configuration from attempt 2 shows faster convergence on first iterations, and the configuration from attempt 6 turns out to have better convergence at the last iterations on 2 of 6 datasets.

**Supplementary Fig. S4**. Boxplots of the eventual log-likelihoods and number of evaluations required for full runs of the genetic algorithm with 6 configurations using the *moments* engine. For each dataset, 2 plots are presented: (1) the top plot shows distribution of 128 resulting log-likelihood values; (2) the bottom plot corresponds to the distribution of the evaluations’ number required for genetic algorithms to terminate. Orange line on boxplot refers to the median value; green triangle demonstrates the mean value.

**Supplementary Fig. S5**. For each dataset, the performance of the new configurations from attempts 2 to 6 is categorized as better, worse, or incomparable to the performance of the default configuration when using the *moments* engine. The histogram illustrates the count of datasets falling into each category for each configuration. Performance is considered better if both the median value and both quartiles of log-likelihoods are higher than those of the default configuration. Conversely, if both the median and quartiles are lower, the dataset is categorized as worse. Otherwise, the comparison is considered similar or undefined.

**Supplementary Fig. S6**. Convergence plots for 6 genetic algorithm configurations using the ∂a∂i engine on 4 training datasets: (1) the default genetic algorithm from the initial version of GADMA and (2–6) configurations obtained during attempts 2 to 6 of hyperparameter optimization with SMAC. The abscissa presents the log-likelihood evaluation number; the ordinate refers to the distance to the optimal value of the log-likelihood. Solid lines correspond to median convergence over 128 runs, and shadowed areas are ranges between first (0.25) and third (0.75) quartiles. The vertical dashed black line refers to the number of evaluations used to stop a genetic algorithm in SMAC.

**Supplementary Fig. S7**. Convergence plots for 6 genetic algorithm configurations on 6 test datasets: (1) the default genetic algorithm from the initial version of GADMA (red) and (2–6) configurations obtained during attempts 2 to 6 of hyperparameter optimization with SMAC.

**Supplementary Fig. S8**. Convergence plots for 6 genetic algorithm configurations using the *momi2* engine on 4 training datasets: (1) the default genetic algorithm from the initial version of GADMA (red) and (2–6) configurations obtained during attempts 2 to 6 of hyperparameter optimization with SMAC. The abscissa presents the log-likelihood evaluation number; the ordinate refers to the distance to the optimal value of log-likelihood. Solid lines correspond to median convergence over 128 runs, and shadowed areas are ranges between first (0.25) and third (0.75) quartiles. The vertical dashed black line refers to the number of evaluations used to stop a genetic algorithm in SMAC.

**Supplementary Fig. S9**. Convergence plots for 6 genetic algorithm configurations on 4 test datasets: (1) default genetic algorithm from the initial version of GADMA (red) and (2–6) configurations obtained during attempts 2 to 6 of hyperparameter optimization with SMAC. Two datasets (2_ButAllA_3_McC, 2_ButSynB2_5_McC) were excluded as they are not supported by the *momi2* engine.

**Supplementary Fig. S10**. Boxplots of the eventual log-likelihoods and number of evaluations required for full runs of the genetic algorithm with 6 configurations using the *momi2* engine. For each dataset, 2 plots are presented: (1) the top plot shows distribution of 128 resulting log-likelihood values; (2) the bottom plot corresponds to the distribution of the evaluations’ number required for genetic algorithms to terminate. Orange line on boxplot refers to the median value; green triangle demonstrates the mean value.

**Supplementary Fig. S11**. For each dataset, the performance of the new configurations from attempts 2 to 6 is categorized as better, worse, or incomparable to the performance of the default configuration when using the *momi2* engine. The histogram illustrates the count of datasets falling into each category for each configuration. Performance is considered better if both the median value and both quartiles of log-likelihoods are higher than those of the default configuration. Conversely, if both the median and quartiles are lower, the dataset is categorized as worse. Otherwise, the comparison is considered similar or undefined.

**Supplementary Fig. S12**. Comparison of the original demographic history of *Drosophila melanogaster* and approximations for the DROS-STRUCT-NOMIG model by *moments* and *moments*LD engines.

**Supplementary Table S1**. Short descriptions of GADMA genetic algorithm (GA) hyperparameters.

**Supplementary Table S2**. Mean log-likelihood values (128 runs) for final configurations of 6 SMAC attempts on training and test datasets. Genetic algorithm was stopped at the same number of evaluations used in SMAC. Log-likelihood was evaluated with the *moments* engine. Mean cost value on training datasets presented in the table is SMAC score that was used by the SMAC intensification procedure. For attempt 1, SMAC failed to find a better configuration than the default one. Best mean values are marked bold.

**Supplementary Table S3**. Mean log-likelihood values (128 runs) for final configurations of 6 SMAC attempts on training and test datasets using the ∂a∂i simulation engine. Genetic algorithm was stopped at the same number of evaluations used in SMAC. Best mean values are marked bold. Log-likelihood values and results are similar to the *moments* engine.

**Supplementary Table S4**. Mean log-likelihood values (128 runs) for final configurations of 6 SMAC attempts on training and test datasets using the *momi2* simulation engine. Genetic algorithm was stopped at the same number of evaluations used in SMAC. Two test datasets (2_ButAllA_3_McC, 2_ButSynB2_5_McC) were excluded as they lacked sequence length required for the *momi2* engine. Moreover, the *momi2* engine does not support continuous migrations, and size of ancestral population could not be inferred implicitly as for ∂a∂i and *moments*. Thus, number of parameters in datasets for *momi2* differs from *moments* and ∂a∂i. Best mean values are marked bold.

**Supplementary Table S5**. Models of *Drosophila melanogaster* populations’ history and GADMA2’s likelihood engines used for performance comparison. If engine was used to infer model parameters, then notation “+” is set between the engine and model; otherwise, notation “–” is specified. Engine *momi2* is not compared for the models with migration as it does not support continuous migrations.

**Supplementary Table S6**. The demographic parameters of *Drosophila melanogaster* history with migration (DROS-MIG model) inferred with different engines in GADMA2. Ground truth includes the parameter values from the original study by Li and Stephan (2006) used in simulation powered by *stdpopsim* (Adrion et al., 2020). Engine *momi2* is excluded as it does not support continuous migrations. Log-likelihood values are not comparable between different engines.

**Supplementary Table S7**. The demographic parameters of *Drosophila melanogaster* history without migration for structure (2, 1) (DROS-STRUCT-NOMIG model) inferred with different engines in GADMA2. Ground truth includes the parameter values from the original study by Li and Stephan (2006) used in simulation powered by stdpopsim (Adrion et al., 2020). Log-likelihood values are not comparable between different engines.

**Supplementary Table S8**. The demographic parameters of *Drosophila melanogaster* history with migration for structure (2, 1) (DROS-STRUCT-MIG model) inferred with different engines in GADMA2. Ground truth includes the parameter values from the original study by Li and Stephan (2006) used in simulation powered by stdpopsim (Adrion et al., 2020). Engine *momi2* is excluded as it does not support continuous migrations. Log-likelihood values are not comparable between different engines.

**Supplementary Table S9**. Comparison of average times for log-likelihood evaluation between used GADMA2’s likelihood engines and models of *Drosophila melanogaster* history. For each model and likelihood engine, mean value and standard deviation are reported.

**Supplementary Table S10**. Mean number of log-likelihood evaluations required for demographic inference averaged over 8 GADMA2 runs for each engine and model of *Drosophila melanogaster* history.

**Supplementary Table S11**. The demographic parameters of *Drosophila melanogaster* history without migration for structure (2, 1) and restriction on population sizes of ancestral and African populations (DROS-STRUCT-NOMIG-AFR model) inferred with different AFS-based engines in GADMA2. Ground truth includes the parameter values from the original article by Li and Stephan (2006) used in simulation powered by stdpopsim (Adrion et al., 2020). Log-likelihood values are not comparable between different engines.

**Supplementary Table S12**. The demographic parameters of *Drosophila melanogaster* history without migration for structure (2, 1) and restrictions on instantaneous expansion of ancestral population and on population sizes of ancestral and African populations (DROS-STRUCT-NOMIG-AFR-ANC model) inferred with different AFS-based engines in GADMA2. Ground truth includes the parameter values from the original study by Li and Stephan (2006) used in simulation powered by stdpopsim (Adrion et al., 2020). Log-likelihood values are not comparable between different engines.

**Supplementary Table S13**. Models of orangutan’s history and GADMA2’s likelihood engines used for performance comparison. If engine was used to infer model parameters, then notation “+” is set between these engine and model; otherwise, notation “−” is specified. Engine *momi2* is not compared for the models with migration as it does not support continuous migrations.

**Supplementary Table S14**. The demographic parameters of orangutan history without migration (ORAN-NOMIG model) inferred with different engines in GADMA2. Ground truth includes the simulated parameter values that were obtained from the original study by Locke et al. (2011).

**Supplementary Table S15**. The demographic parameters of orangutan history with migration (ORAN-MIG model) inferred with different engines in GADMA2. Ground truth includes the simulated parameter values that were obtained from the original study by Locke et al. (2011). *Momi2* engine was excluded as it does not support continuous migrations.

**Supplementary Table S16**. The demographic parameters of orangutan history without migration for structure (1, 1) (ORAN-STRUCT-NOMIG model) inferred with different engines in GADMA2. Ground truth includes the simulated parameter values that were obtained from the original study by Locke et al. (2011). Log-likelihood values are not comparable between different engines.

**Supplementary Table S17**. The demographic parameters of orangutan history for several models without migrations inferred using the *moments* engine in GADMA2. Models differ by set of dynamics used for inference and by the restriction on population sizes. Model follows the restriction (marked by +) when the sum of Bornean and Sumatran population sizes is obliged to equal the ancestral population size before the split. Two histories are also presented in another table that are indicated in the last row. Ground truth includes the simulated parameter values that were obtained from the original study by Locke et al. (2011).

**Supplementary Table S18**. The demographic parameters of orangutan history for models with linear size change of Sumatran population and without migrations inferred with different engines in GADMA2. Ground truth includes the simulated parameter values that were obtained from the original study by Locke et al. (2011). *Momi2* engine was excluded as it does not support linear size change. Log-likelihood values are not comparable between different engines.

**Supplementary Table S19**. Comparison of average times for log-likelihood evaluation between used GADMA2’s likelihood engines and models of orangutan history. For each model and likelihood engine, mean value and standard deviation are reported.

**Supplementary Table S20**. Mean number of log-likelihood evaluations required for demographic inference averaged over 8 GADMA2 runs for each engine and model of orangutan history.

**Supplementary Table S21**. Result statistics obtained from 100 repeats of 2 of ∂a∂i’s optimization techniques and GADMA2 in a case of the demographic inference without inbreeding for the American Puma populations. The reported statistics include the mean and standard deviation of the number of evaluations, CPU times, and log-likelihoods. GADMA2 is compared with 2 of ∂a∂i’s optimization techniques: single optimization and optimization with multiple restarts. In order to match the number of evaluations with GADMA2, the ∂a∂i optimization with multiple restarts has 18 restarts. Additionally, results from Blischak et al. (2020) are included, which were obtained using single ∂a∂i optimization with different initialization process. BFGS optimization was used as an optimization from ∂a∂i. The results obtained from GADMA2 are highlighted in bold, as they achieved the best mean and best log-likelihood values.

**Supplementary Table S22**. Result statistics obtained from 100 repeats of 2 of ∂a∂i’s optimization techniques and GADMA2 in a case of the demographic inference with inbreeding for the American Puma populations. The reported statistics include the mean and standard deviation of the number of evaluations, CPU times, and log-likelihoods. GADMA2 is compared with 2 of ∂a∂i’s optimization techniques: single optimization and optimization with multiple restarts. In order to match the number of evaluations with GADMA2, the ∂a∂i optimization with multiple restarts has 16 restarts. Additionally, results from Blischak et al. (2020) are included, which were obtained using single ∂a∂i optimization with different initialization process. BFGS optimization was used as an optimization from ∂a∂i. The results obtained from GADMA2 are highlighted in bold, as they achieved the best mean and best log-likelihood values.

**Supplementary Table S23**. Log-scale standard deviations for parameters in the model without inbreeding for American pumas across a series of step sizes.

**Supplementary Table S24**. Log-scale standard deviations for parameters in the model with inbreeding for American pumas across a series of step sizes.

**Supplementary Table S25**. Maximum likelihood parameters inferred from the demographic models for the Texas and Florida populations of American puma.

**Supplementary Table S26**. Result statistics obtained from 100 repeats of 2 of ∂a∂i’s optimization techniques and GADMA2 in a case of the demographic inference without inbreeding for the domesticated cabbage. The reported statistics include the mean and standard deviation of the number of evaluations, CPU times, and log-likelihoods. GADMA2 is compared with 2 of ∂a∂i’s optimization techniques: single optimization and optimization with multiple restarts. Additionally, results from Blischak et al. (2020) are included, which were obtained using single ∂a∂i optimization with a different initialization process. In order to match the number of evaluations with GADMA2, the ∂a∂i optimization with multiple restarts has 27 restarts. BOBYQA optimization was used as an optimization from ∂a∂i. The results obtained from ∂a∂i’s optimization with multiple restarts are highlighted in bold, as they achieved the best mean and best log-likelihood values.

**Supplementary Table S27**. Result statistics obtained from 100 repeats of 2 of ∂a∂i’s optimization techniques and GADMA2 in a case of the demographic inference with inbreeding for the domesticated cabbage. The reported statistics include the mean and standard deviation of the number of evaluations, CPU times, and log-likelihoods. GADMA2 is compared with 2 of ∂a∂i’s optimization techniques: single optimization and optimization with multiple restarts. Additionally, results from Blischak et al. (2020) are included, which were obtained using single ∂a∂i optimization with a different initialization process. In order to match the number of evaluations with GADMA2, the ∂a∂i optimization with multiple restarts has 16 restarts. BOBYQA optimization was used as an optimization from ∂a∂i. The results obtained from ∂a∂i’s optimization with multiple restarts are highlighted in bold, as they achieved the best mean and slightly better than GADMA2 best log-likelihood values.

**Supplementary Table S28**. Log-scale standard deviations for parameters in the model without inbreeding for domesticated cabbage across a series of step sizes.

**Supplementary Table S29**. Log-scale standard deviations for parameters in the model with inbreeding for domesticated cabbage across a series of step sizes.

**Supplementary Table S30**. Maximum likelihood parameters inferred from the demographic models for the domesticated cabbage population.

## Abbreviations

AFS: allele frequency spectrum; bp: base pair; CI: confidence interval; Gbp: gigabase pair; LD: linkage disequilibrium.

## References

[1] Der Sarkissian C, Ermini L, Schubert M et al. Evolutionary genomics and conservation of the endangered Przewalski’s horse. Curr Biol. 2015;25(19):2577–83. 26412128 10.1016/j.cub.2015.08.032 PMC5104162

[2] Abascal F, Corvelo A, Cruz F, et al. Extreme genomic erosion after recurrent demographic bottlenecks in the highly endangered Iberian lynx. Genome Biol. 2016;17(1):1–19. 27964752 10.1186/s13059-016-1090-1 PMC5155386

[3] Payet SD, Pratchett MS, Saenz-Agudelo P et al. Demographic histories shape population genomics of the common coral grouper (Plectropomus leopardus). Evol Appl. 2022;15(8):1221–35. 36051464 10.1111/eva.13450 PMC9423088

[4] Gutenkunst RN, Hernandez RD, Williamson SH, et al. Inferring the joint demographic history of multiple populations from multidimensional SNP frequency data. PLoS Genet. 2009;5(10):e1000695. 19851460 10.1371/journal.pgen.1000695 PMC2760211

[5] Gronau I, Hubisz MJ, Gulko B et al. Bayesian inference of ancient human demography from individual genome sequences. Nat Genet. 2011;43(10):1031–4. 21926973 10.1038/ng.937 PMC3245873

[6] Cornuet JM, Pudlo P, Veyssier J et al. DIYABC v2. 0: a software to make approximate Bayesian computation inferences about population history using single nucleotide polymorphism, DNA sequence and microsatellite data. Bioinformatics. 2014;30(8):1187–9. 24389659 10.1093/bioinformatics/btt763

[7] Jouganous J, Long W, Ragsdale AP et al. Inferring the joint demographic history of multiple populations: beyond the diffusion approximation. Genetics. 2017;206(3):1549–67. 28495960 10.1534/genetics.117.200493 PMC5500150

[8] Hey J, Chung Y, Sethuraman A et al. Phylogeny estimation by integration over isolation with migration models. Mol Biol Evol. 2018;35(11):2805–18. 30137463 10.1093/molbev/msy162 PMC6231491

[9] Steinrücken M, Kamm J, Spence JP et al. Inference of complex population histories using whole-genome sequences from multiple populations. Proc Natl Acad Sci U S A. 2019;116(34):17115–20. 31387977 10.1073/pnas.1905060116 PMC6708337

[10] Kamm J, Terhorst J, Durbin R et al. Efficiently inferring the demographic history of many populations with allele count data. J Am Stat Assoc. 2020;115(531):1472–87. 33012903 10.1080/01621459.2019.1635482 PMC7531012

[11] Excofffier L, Marchi N, Marques DA, et al. fastsimcoal2: demographic inference under complex evolutionary scenarios. Bioinformatics. 2021;37(24):4882–85. 34164653 10.1093/bioinformatics/btab468 PMC8665742

[12] DeWitt WS, Harris KD, Ragsdale AP, et al. Nonparametric coalescent inference of mutation spectrum history and demography. Proc Natl Acad Sci U S A. 2021;118(21):e2013798118 34016747 10.1073/pnas.2013798118 PMC8166128

[13] Ragsdale AP, Gutenkunst RN. Inferring demographic history using two-locus statistics. Genetics. 2017;206(2):1037–48. 28413158 10.1534/genetics.117.201251 PMC5499162

[14] Ragsdale AP, Gravel S. Models of archaic admixture and recent history from two-locus statistics. PLoS Genet. 2019;15(6):e1008204. 31181058 10.1371/journal.pgen.1008204 PMC6586359

[15] Ragsdale AP, Gravel S. Unbiased estimation of linkage disequilibrium from unphased data. Mol Biol Evol. 2020;37(3):923–32. 31697386 10.1093/molbev/msz265 PMC7038669

[16] Noskova E, Ulyantsev V, Koepfli KP, et al. GADMA: genetic algorithm for inferring demographic history of multiple populations from allele frequency spectrum data. Gigascience. 2020;9(3):giaa005. 32112099 10.1093/gigascience/giaa005 PMC7049072

[17] Xiong P, Hulsey CD, Fruciano C et al. The comparative genomic landscape of adaptive radiation in crater lake cichlid fishes. Mol Ecol. 2021;30(4):955–72. 33305470 10.1111/mec.15774 PMC8607476

[18] Valdez L, D’Elía G. Genetic diversity and demographic history of the shaggy soft-haired mouse abrothrix hirta (Cricetidae; Abrotrichini). Front Genet. 2021;12:184. 10.3389/fgene.2021.642504 PMC8024643 33841502

[19] Pazhenkova EA, Lukhtanov VA. Genomic introgression from a distant congener in the Levant fritillary butterfly, Melitaea acentria. Mol Ecol. 2021;30(19):4819–32. 34288183 10.1111/mec.16085

[20] Cassin-Sackett L, Campana MG, McInerney NR et al. Genetic structure and population history in two critically endangered Kaua ‘i honeycreepers. Conserv Genet. 2021:22(4):601–14.

[21] Buggiotti L, Yurchenko AA, Yudin NS et al. Demographic history, adaptation, and NRAP convergent evolution at amino acid residue 100 in the world northernmost cattle from Siberia. Mol Biol Evol. 2021;38(8):3093–3110. 33784744 10.1093/molbev/msab078 PMC8321547

[22] Blischak PD, Barker MS, Gutenkunst RN. Inferring the demographic history of inbred species from genome-wide SNP frequency data. Mol Biol Evol. 2020;37(7):2124–36. 32068861 10.1093/molbev/msaa042 PMC7828618

[23] Gutenkunst RN . dadi. CUDA: accelerating population genetics inference with graphics processing units. Mol Biol Evol. 2021;38(5):2177–8. 33480999 10.1093/molbev/msaa305 PMC8097298

[24] Noskova E . Package deminf_data. GitHub. https://github.com/noscode/demographic_inference_data.

[25] Adrion JR, Cole CB, Dukler N et al. A community-maintained standard library of population genetic models. Elife. 2020;9:e54967. 32573438 10.7554/eLife.54967 PMC7438115

[26] Kelleher J, Etheridge AM, McVean G. Efficient coalescent simulation and genealogical analysis for large sample sizes. PLoS Comput Biol. 2016;12(5):e1004842. 27145223 10.1371/journal.pcbi.1004842 PMC4856371

[27] Li H, Stephan W. Inferring the demographic history and rate of adaptive substitution in Drosophila. PLoS Genet. 2006;2:(10):e166. 17040129 10.1371/journal.pgen.0020166 PMC1599771

[28] Schrider DR, Houle D, Lynch M, et al. Rates and genomic consequences of spontaneous mutational events in Drosophila melanogaster. Genetics. 2013;194(4):937–54. 23733788 10.1534/genetics.113.151670 PMC3730921

[29] Comeron JM, Ratnappan R, Bailin S. The many landscapes of recombination in Drosophila melanogaster. PLoS Genet. 2012;8(10):1–21. 10.1371/journal.pgen.1002905 PMC3469467 23071443

[30] Locke DP, Hillier LW, Warren WC et al. Comparative and demographic analysis of orangutan genomes. Nature. 2011;469(7331):529–33. 21270892 10.1038/nature09687 PMC3060778

[31] Nater A, Mattle-Greminger MP, Nurcahyo A et al. Morphometric, behavioral, and genomic evidence for a new orangutan species. Curr Biol. 2017;27(22):3487–98. 29103940 10.1016/j.cub.2017.09.047

[32] Ochoa A, Onorato DP, Fitak RR et al. De novo assembly and annotation from parental and F1 puma genomes of the Florida panther genetic restoration program. G3 (Bethesda). 2019;9(11):3531–6. 31519748 10.1534/g3.119.400629 PMC6829145

[33] Cheng F, Wu J, Cai C et al. Genome resequencing and comparative variome analysis in a Brassica rapa and Brassica oleracea collection. Sci Data. 2016;3(1):1–9. 10.1038/sdata.2016.119 PMC5170593 27996963

[34] Cheng F, Sun R, Hou X et al. Subgenome parallel selection is associated with morphotype diversification and convergent crop domestication in Brassica rapa and Brassica oleracea. Nat Genet. 2016;48(10):1218–24. 27526322 10.1038/ng.3634

[35] Blischak PD, Barker MS, Gutenkunst RN. Data and results for “Inferring the Demographic History of Inbred Species from Genome-Wide SNP Frequency Data.”. *GitHub*. https://github.com/pblischak/inbreeding-sfs. 10.1093/molbev/msaa042 PMC7828618 32068861

[36] Snoek J, Larochelle H, Adams RP. Practical bayesian optimization of machine learning algorithms. Adv Neural Inform Process Syst. 2012;25.

[37] Hutter F, Hoos HH, Leyton-Brown K. Sequential model-based optimization for general algorithm configuration. In: International Conference on Learning and Intelligent Optimization. Berlin Heidelberg: Springer; 2011:507–23.

[38] Lindauer M, Eggensperger K, Feurer M, et al. SMAC3: a versatile bayesian optimization package for hyperparameter optimization. J Machine Learn Res. 2022;23(54):1–9.

[39] Lago J, De Ridder F, Vrancx P, et al. Forecasting day-ahead electricity prices in Europe: the importance of considering market integration. Appl Energ. 2018;211:890–903.

[40] Hewamalage H, Bergmeir C, Bandara K. Recurrent neural networks for time series forecasting: current status and future directions. Int J Forecasting. 2021;37(1):388–427.

[41] Wu S, Song X, Feng Z, et al. NFLAT: non-flat-lattice transformer for chinese named entity recognition. arXiv preprint arXiv:220505832 2022. 10.48550/arXiv.2205.05832.

[42] Coffman AJ, Hsieh PH, Gravel S, et al. Computationally efficient composite likelihood statistics for demographic inference. Mol Biol Evol. 2016;33(2):591–3. 26545922 10.1093/molbev/msv255 PMC5854098

[43] Broyden CG . The convergence of a class of double-rank minimization algorithms 1. general considerations. IMA J Appl Math. 1970;6(1):76–90.

[44] Fletcher R . A new approach to variable metric algorithms. Comput J. 1970;13(3):317–22.

[45] Goldfarb D . A family of variable-metric methods derived by variational means. Math Comput. 1970;24(109):23–6.

[46] Shanno DF . Conditioning of quasi-Newton methods for function minimization. Math Comput. 1970;24(111):647–56.

[47] Powell MJ . The BOBYQA algorithm for bound constrained optimization without derivatives. Cambridge NA Report NA2009/06. Cambridge, UK: University of Cambridge;. 2009.

[48] Williamson SH, Hernandez R, Fledel-Alon A et al. Simultaneous inference of selection and population growth from patterns of variation in the human genome. Proc Natl Acad Sci U S A. 2005;102(22):7882–7. 15905331 10.1073/pnas.0502300102 PMC1142382

[49] Overcast I . EasySFS. *GitHub*. https://github.com/isaacovercast/easySFS.

[50] Myers S, Fefferman C, Patterson N. Can one learn history from the allelic spectrum?. Theor Popul Biol. 2008;73(3):342–8. 18321552 10.1016/j.tpb.2008.01.001

[51] Gower GR, Ragsdale AP, Gutenkunst RN et al. Demes: a standard format for demographic models. Genetics. 2022;222(3):iyac131. 10.1093/genetics/iyac131. 36173327 PMC9630982

[52] Eyre-Walker A, Keightley PD. The distribution of fitness effects of new mutations. Nat Rev Genet. 2007;8(8):610–8. 17637733 10.1038/nrg2146

[53] Noskova E, Abramov N, Iliutkin S, et al. GADMA2 documentation. https://gadma.readthedocs.io. Accessed 25 July 2023.

[54] Rosenbrock H . An automatic method for finding the greatest or least value of a function. Comput J. 1960;3(3):175–84.

[55] Noskova E, Abramov N, Iliutkin S et al. Supporting data for “GADMA2: More Efficient and Flexible Demographic Inference from Genetic Data.”. GigaScience Database. 2023. 10.5524/102403. PMC10445054 37609916

[56] Noskova E . Results of hyperparameter optimization for “GADMA2: more efficient and flexible demographic inference from genetic data.”. *GitHub*. https://github.com/noscode/HPO_results_GADMA. 10.1093/gigascience/giad059 PMC10445054 37609916

[57] Noskova E, Ulyantsev V, Koepfli KP, et al. Data and results for “GADMA: genetic algorithm for inferring demographic history of multiple populations from allele frequency spectrum data.”. https://bitbucket.org/noscode/gadma_results. 10.1093/gigascience/giaa005 PMC7049072 32112099

