# Supplementary material for: GADMA2: more efficient and flexible demographic inference from genetic data
Source: Gigascience. 2023 Aug 23;12:giad059. doi: 10.1093/gigascience/giad059 (PMC10445054; doi:10.1093/gigascience/giad059)
Supplement: giad059_Response_to_Reviewer_Comments_Original_Submission [file giad059_response_to_reviewer_comments_original_submission.pdf]

Author's Response To Reviewer Comments

Close

Dear Editor and Reviewers,

We are grateful for the reviewers' constructive feedback and suggestions, which have helped us to enhance the quality of our manuscript. We have carefully considered all comments and made revisions to the manuscript and supplementary materials. The changes are highlighted in blue for easy reference. A brief summary of the main changes is provided below.

Main changes:

- The term “inference engine” changed to “likelihood engine”
- Updated comparison of five new configurations obtained from hyperparameter optimizations with SMAC:
* Added more details about SMAC score (section S1.1 and “Materials and methods”)
* Reported SMAC scores averaged over datasets in Tables S2, S3, and S4
* Compared full runs of genetic algorithm with different configurations
* Added Figures S4 and S9 with boxplots of log-likelihoods and evaluations for full run of each configuration
* Added sections S1.2 and S1.3 with detailed comparison
* Summarized GADMA2 improvement in Figure 3
- Merged Table S2 into Table 1 as suggested by Reviewer 2
- Compared two result histories of fruit fly with different metrics as suggested by Reviewer 2:
* Added section S2.1.1 with details and added results in the manuscript
- Performed additional experiment for orangutan simulated dataset for model without restriction on population sizes:
* Updated Section S2.2 and added Table S17
- Compared GADMA2 with dadi optimization techniques regarding the comment of Reviewer 1:
* Removed Figure 4 and Figure S9
* Ran dadi optimization multiple times to attain computational costs of GADMA2
* Included Tables S20, S21 for American puma dataset and Tables S25, S26 for domesticated cabbage dataset with results of comparison
* Added description and results in "Materials and Methods" and "Results and Discussion" sections
- Reran experiments for "Inference with inbreeding" section with correct hyperparameters:
* Updated Tables S24 and S29
- Evaluated confidence intervals with different step sizes as suggested by Reviewer 1:
* Added new Tables S22, S23, S27, and S28.

A point-by-point response to the comments can be found below.

Reviewer 1
==========

In this paper, the authors present GADMA 2, an update of their population genomic inference software GADMA. The author's software serves as a driver for other population genomics software, enabling a consistent user interface and a different parameter optimization approach. GADMA 2 extends GADMA by adding two new inference engines: momi2 and momentsLD, hyperparameter optimization for the genetic algorithm, demes visualization, selection, dominance, and inbreeding modeling, and a new method for specifying model structures. In this paper, the authors show that their optimized genetic algorithm is somewhat more effective than the original hyperparameter settings. They also compare among inference engines, finding some differences in behavior. Lastly they compare with dadi itself in two models with

inbreeding, finding better likelihood parameter sets than those previously published.

GADMA has already found some use in the population genomics community, and GADMA 2 is a substantial update. The manuscript describes the updates in good detail and demonstrates the effectiveness of GADMA 2 on two real-world data sets. Overall, this is a strong contribution, and we have few major concerns.

>>> Thank you very much! We have addressed each of the comments in turn below.

Major Technical Concerns
----

1) The authors claim to now support inference of selection and dominance. But what they support is very limited and not very biological. In particular, they currently support inferences which assume a single selection and dominance coefficient for the entire data set (as in Williamson et al. (2005) PNAS). In reality, any AFS will include sites with a variety of selection coefficients, usually summarized by a distribution of fitness effects. Since Keightley and Eyre-Walker (2007) Genetics, this has been the standard for inferring selection from the AFS. The authors should be clear about the limitations of what they have implemented.

>>> We are grateful for bringing this important point to our attention. We have added a clarification of the approach limitations in paragraph 3 of the section "Flexible structure model" on page 6 and in paragraph 3 of the section "Conclusions" on page 9 of the manuscript. We hope that these additions help to provide a clearer understanding of the current capabilities of the included approach.

2) Figure 4 shows that optimization runs using GADMA 2 tend to find better likelihoods than bare dadi optimization runs. But the advice for using dadi or moments is to run multiple optimizations and take the best likelihood found, with some heuristic for assessing convergence. So most users would not (or at least should not) stop with the result of a single dadi optimization run. It does seem that GADMA 2 reduces the complexity of assessing convergence between multiple dadi optimization runs. But another important consideration is computational cost. (At an extreme, if each dadi run was 100 times faster than a single GADMA 2 run, then the correct comparison would be between the best of 100 dadi runs and a single GADMA 2 run.) It is not clear from the paper how the 100 GADMA 2 runs compare to the 100 dadi runs in terms of computational cost. It would be very helpful to have a table or some text describing the average computational cost (in CPU hours) of those runs.
Thank you for pointing out the importance of considering computational costs when comparing optimization methods. We have taken this into account and have added new results to our manuscript that compare GADMA2 and dadi optimizations in terms of the computational costs.
We reran experiments from Blischak et al., 2020. We compared GADMA2 and dadi optimization with multiple repeats. Number of the repeats was chosen to match computational costs of GADMA2. We consider that the number of evaluations gives a better indication of the computational cost than a CPU time because the evaluation time of the log-likelihood function is not constant and changes depending on the parameters values during optimization. The results of our experiments show that GADMA2 has better performance than single dadi optimization. However, dadi optimization with repeats can sometimes yield better results than GADMA2. Despite these, GADMA2 still has an advantage of reducing complexity of the demographic inference process.

>>> We have emphasized these findings in the relevant sections of the manuscript, including "Performance test of GADMA2 engines" (paragraph 3 of the section, page 4), "Usage case: inference of inbreeding coefficients" (paragraph 2, page 8), and "Conclusions" (paragraph 8, page 9). In addition, we have added tables with the results of our runs in the supplementary materials (Tables S20, S21, S25, S26). We have also removed Figures 4 and Figure S9.

Major Writing / Presentation Concerns:
----

1) Bottom of page 5: The authors are sharing the results of their hyperparameter optimizations from their own server, with uncertain lifetime. These results should be moved to an archival service such as Dryad.

>>> Thank you very much for this concern, it was our initial solution of storage. As the Editor suggested, we will store the results of our hyperparameter optimizations in GigaDB, an archival service that ensures long-term access to data.

Minor Technical Concerns:
----

1) The authors note that the DROS-MIG models had worse likelihoods than the DROS-NOMIG models. Since these are nested models, the DROS-MIG model must mathematically have a better global optimum likelihood. It would be worth pointing out that the likelihoods they found indicate a failure of the optimization algorithms. The authors should also present the DROS-MIG model results in a supplementary table.

>>> We have added the note about optimization failure in the manuscript (paragraph 1, section “Fruit fly demographic history”, page 7). Table with DROS-MIG model results is included as Table S6.

2) The Godambe parameter uncertainties in Tables S20 and S21 are pretty extreme, sometimes 10^-13 to 10^12. This may be due to instability of the Godambe approximation versus step size. In Blischak et al. (2020) Mol Biol Evol, the authors tried several step sizes and sought consistent results between them (Tables S1-S4). We suggest the authors take that approach here.

>>> We have taken the suggestion into consideration and have applied the approach from Blischak et al. (2020) to evaluate confidence intervals. Despite using different step sizes, we still observed significant uncertainties. We have added new tables (Tables S20, S21 for puma and Tables S23, S24 for cabbage) to our manuscript to reflect these results.

Minor Writing / Presentation Concerns:
----

1) The author claims that "GADMA does not require model specification". However, it seems that GADMA "structure model" rather describes a different and perhaps broader way to specify demographic models rather than completely eliminates model specification.

>>> You are totally correct. GADMA’s structure model is an extended model that includes dynamics of population sizes as discrete parameters. However, such a model is determined by its structure and is more easy-to-set for GADMA. It means that GADMA does not require detailed/complex model specification in a sense of other classical tools like dadi or moments. We have added an adjective “complicated” in the sentence for clarity (page 2).

2) The authors use the term "inference engine" for the four tools GADMA 2 builds upon. But to us, the act of inference includes parameter optimization. In this case, these tools are not being used for the inference itself, but rather to calculate the (composite) likelihood of the data. Perhaps a better term would be "likelihood calculator"?

>>> That is a very valuable suggestion. We had several variants about the term that denotes the engine of GADMA and several concerns about each of them as well. Our vision is that the term must include the word “engine” as it is something that runs the whole GADMA. We have changed “inference engine” to “likelihood engine” throughout the manuscript and Supplementary materials.

3) The authors suggest engine-specific hyperparameter optimization as a future goal. But the optimal hyperparameters are also likely to be model specific. (For example, 2- versus 4-population models might benefit from different optimization regimes.) Can the authors comment on this?

>>> Thank you very much for that very interesting suggestion for future work. We have added this proposal and highlighted its benefits in paragraph 2 of section “Conclusions” on page 9 of our manuscript.

Writing Nitpicks

----

1) Abstract: "optimization algorithms used to find model parameters sometimes turn out to be inefficient" → vague: more details on why/how they are inefficient would be helpful
2) Introduction: "Inference of complex demographic histories… in the population's past." needs citation.
3) Page 2: "parameter to infer, for example, all migration" is a comma splice and should be split into two sentences.
4) Supplement page 4: Figure ?? reference is broken.

>>> Thank you very much, everything was added and fixed.

Reviewer 2
==========

This manuscript describes updates made to GADMA, which was published two years ago. GADMA uses likelihood-based demography inference methods as likelihood-computation engines, and replaces their generic optimization technique with a more sophisticated technique based on a genetic algorithm. The version of GADMA described in this manuscript has several important added features. It supports two additional inference engines, more flexible models, additional input and output formats, and it provides better values for the hyper-parameters used by the genetic algorithm. This is indeed a substantial improvement over the original version of GADMA. The manuscript clearly describes the different added features to GADMA, and then demonstrates them with a series of analyses. These analyses establish three main things: (1) they show that the new hyper-parameters improve performance; (2) they show how GADMA can be used to compare performance of different approaches to calculate data likelihood for demography inference; (3) showcase new features of GADMA (supporting model structure and inbreeding inference). Overall, the presentation is very clear and the results are interesting and compelling. Thus, despite being a publication about a method update, it shows substantial improvement, provides interesting new insights, and will likely lead to expansion of the user base for GADMA.

>>> We would like to express our gratitude to the Reviewer for his support and comments.

The only major comment I have is about the part of the study that optimizes the hyper-parameters. The hyper-parameter optimization is a very important improvement in GADMA2. The setup for this analysis is very good, with three inference engines, four data sets used for training and six diverse data sets used for testing. However, because of complications with SMAC for discrete hyper-parameters, the analysis ends up considering six separate attempts. The comparison between the hyper-parameters produced by these six attempts is mostly done manually across data sets and inference engines. This somewhat beats the purpose of the well-designed set up. Eventually, it is very difficult for the reader to asses the expected improvement of the final suggested values of hyper-parameters (attempt 2) to the default ones. I have two comments/suggestions about this part.

>>> We have addressed each of the comments in turn below.

First, I'm wondering if there is a formal way to compare the eventual parameters of the six attempts across the four training sets. I can see why you would need to run SMAC six separate times to deal with the discrete parameters. However, why do you not use the SMAC score to compare the final settings produced by these six runs?

>>> There are two reasons why we do not use SMAC scores to compare final configurations. First, the SMAC score is the average log-likelihood obtained by the genetic algorithm within a fixed number of evaluations, which does not necessarily reflect the overall convergence of the algorithm. Additionally, log-likelihoods have different scales between datasets (e.g. log-likelihood of ~90 for one population and of ~11,000 for three populations), so it is not appropriate to average uneven values as it is done by SMAC score.
>>> To provide clarity, we have updated the manuscript to include more information about the SMAC

score and elaborate on the reasons above. We have also included a more detailed comparison of the configurations' full runs for each dataset, as well as additional information about SMAC specifications and the target function. We have revised paragraph 4 of the "Hyperparameter Optimization" section (page 3) and paragraphs 2-4 of the "Updated Genetic Algorithm" section (page 5) for this purpose. Additionally, we have formed a new section S1.2 and added a section S1.3 with detailed comparison.

Second, as a reader, I would like to see a single table/figure summarizing the improvement you get using whatever hyper-parameters you end up suggesting in the end compared to the default setting used in GADMA1. This should cover all the inference engines and all the data sets somehow in one coherent table/figure. Using such a table/figure, you could report improvement statistics, such as the average increase in log-likelihood, or average decrease in convergence times. These important results get lost in the many improved figures and tables.

>>> We have included Figure 3 to highlight the improvement in the performance of GADMA2 compared to the original version. Our goal was to clearly demonstrate the speedup of GADMA2 through a bar plot presentation. The figure also provides information on the comparison of log-likelihoods, where the bars are filled with hatching patterns that indicate whether the GADMA2 likelihood is better, worse, or similar to that of GADMA v1. We made a conscious decision not to color the bar plots in order to prevent any misinterpretation, as colors in bar plots are usually associated with the values represented by the bar heights, which is not the case here.

These are my main suggestions for revisions of the current version. I also have some more minor comments that the authors may wish to consider in their revised version, which I list below.

Introduction:
----

para 2: the survey of demography inference methods focuses on likelihood-based methods, but there is a substantial family of Bayesian inference methods, such as MPP, Ima, and G-PhoCS. Bayesian methods solve the parameter estimation problem by Bayesian sampling. I admit that this is somewhat tangential to what GADMA is doing, but this distinction between likelihood-based methods and Bayesian methods probably deserves a brief mention in the introduction.

>>> Thank you very much for pointing out that we failed to mention Bayesian methods in our survey. We have added it in the revised version in paragraph 2 of section “Introduction” on page 1.

para 2,3: you mention a result from the original GADMA paper showing that GADMA improves on the optimization methods implemented by current demography inference methods. Readers of this paper might benefit of a brief summary of the improvement you were able to achieve using the original version of GADMA. Can you add 2-3 sentences providing the highlights of the improvement you were able to show in the first paper?

>>> We have added two sentences about the main results of the initial version of GADMA in paragraph 3 of section “Introduction” on page 2.

para 3: The statement "GADMA separates two regular components" is not very clear. Can you rephrase to clarify?

>>> To avoid confusion of the reader we have removed the sentence "GADMA separates two regular components".

Materials and methods - Hyper-parameter optimization:

I didn't fully understand what you use for the cost function in SMAC here. Seems to me like there are two criteria: accuracy and speed. You wish the final model to be as accurate as possible (high log likelihood), but you want to obtain this result with few optimization iterations. Can you briefly describe how these two

objectives are addressed in your use of SMAC? It's also not completely clear how results from different engines and different data sets are incorporated into the SMAC cost. Can you provide more details about this in the supplement?

>>> We have included paragraph 5 of section S1.1 on page 3 of Supplementary materials with details about SMAC cost function.
>>> Briefly, SMAC can perform a hyperparameters search according to either speed or accuracy criterion. In order to achieve both goals we use accuracy setting but provide genetic algorithms with a fixed number of evaluations. Thus, the genetic algorithm is terminated earlier during the SMAC framework. SMAC uses the cost function (result log-likelihood of early terminated genetic algorithm) averaged over given training datasets as a target function for the hyperparameter optimization. Thus, it is important for training datasets to have comparable log-likelihoods.

para 2: "That eliminate three combinations" should be "This eliminates three combinations".
para 3: "Each attempt is running" should be "Each attempt ran"

>>> Thank you, both were fixed.

para 3: "We take 200×number of parameters as the stop criteria". Can you clarify? Does this mean that you set the number of GADMA iterations to 200 times the number of demographic model parameters? Why should it be a linear function of the number of parameters? The following text explains the justification, but

>>> We have included the clarifications in paragraph 5 of section S1.1 on page 3 of Supplementary materials. The sentence "We take 200×number of parameters as the stop criteria" means that the genetic algorithm is stopped when it attains the number of evaluations equal to 200 times the number of demographic model parameters. The number of evaluations required for convergence is not always linear to the number of parameters. However, we chose it to be linear to provide a sufficient budget for larger datasets.

Table 1: I would merge Table S2 with this one (by adding the ranges of all hyper-parametres as a first column). It's important to see the ranges when examining the different selections.

>>> Thank you, we have merged Table S2 into Table 1.

Materials and methods - Performance test of GADMA2 engines:
----

para 2: "ROS-STRUCT-NOMIG" should be "DROS-STRUCT-NOMIG" Also, "This notation could be read" - maybe replace by "This notation means" to signal that you're explaining the structure notation.

>>> Thank you, we fixed both notes.

Para 4 (describing comparisons for momi on Orangutan data): "ORAN-NOMIG model is compared with three …". You also consider ORAN-STRUCT-NOMIG in the momi analysis, right?

>>> You are totally correct, we missed the ORAN-STRUCT-NOMIG model from the comparison. We have changed the sentence and also added results for the ORAN-STRUCT-NOMIG model in Table 4.

Results - Performance test of GADMA2 engines:

Inference for the Drosophila data set under model with migration: you mention that the models with migration obtain lower likelihoods than the models without migration. You cannot directly compare likelihoods in these two models, since the likelihood surface is not identical. So, I'm not sure that the fact that you get higher likelihoods in the models without migration is a clear enough indication for model fit. The fact that the inferred migration rates are low is a good indication for that. It also seems like despite converging to models with very low migration rates, the other parameters are inferred with higher noise.

For example, the size of the European bottleneck is significantly increased in these inferences compared to that of the NOMIG. So, potentially the problem here is that more time is required for these complex models to converge.

>>> As mentioned in the comment of Reviewer 1 those two models are nested meaning that the model DROS-MIG can achieve the same parameters and log-likelihood as the model DROS-NOMIG. Therefore, we are allowed to compare their log-likelihoods. We have added an additional sentence “Since DROS-NOMIG and DROS-MIG models are nested this indicates optimization failure.” on page 7.
>>> The log-likelihood surfaces can be (and probably are) different between those models, but the purpose of GADMA is to overcome these difficulties automatically. Even though running GADMA for more iterations might improve the result we consider it as a failure as it was not done automatically.

Inference for the Drosophila data set under structured model (2,1): the values inferred by moments and momentsLD appear to neatly fit the true values. However, it is not straightforward to compare an exponential increase in population size to an instantaneous increase. Maybe this can be done by some time-averaged population size, or the average time until coalescence in the two models? This will allow you to quantify how good the two exponential models fit the true model with instantaneous increase.

>>> We are grateful for that suggestion. We compared exponential growth approximations received for moments and momentsLD engines. We used several metrics: mean population size, harmonic mean population size and time until coalescence. The details about used metrics are available in section S2.1.1 on page 20 of Supplementary materials. The conclusions are presented in para 2 of section “Fruit fly demographic history” on page 7.

Inference for the Orangutan data set under structured model (2,1) without migration: you argue that a constant population size is inferred for Bor by moments and momi because of the restriction on population sizes after the split. You base this claim on a comparison between the log-likelihoods obtained in this model (STRUCT-NOMIG) and the standard model (NOMIG) in which you add this restriction. I didn't fully understand how you can conclude from this comparison that the constant size inferred for Bor is due to the restriction on the initial population size after the split. I think what you need to do to establish this is run the STRUCT model without this restriction and see that you get exponential decrease. Can you elaborate more on your rationale? A detailed explanation should appear in the supplement and a brief summary in the main text.

>>> Thank you for bringing to our attention this crucial aspect that was missing from our manuscript! Our original idea was to check that the best log-likelihood for the ORAN-NOMIG model with restriction is worse than the likelihood obtained for the ORAN-STRUCT-NOMIG model (with restriction). However, as you correctly noted, it's difficult to conclude that the constant size of the ORAN-STRUCT-NOMIG model was solely due to the restriction based on this comparison. This result only demonstrates that our histories for the ORAN-STRUCT-NOMIG model are optimal.
>>> We have conducted an experiment for the ORAN-STRUCT-NOMIG model without the restriction, and the results showed a correct exponential population size change for the Bornean population. Therefore, our initial conclusion is still valid. Additionally, we found an unexpected and interesting result: the population size dynamics of the Sumatran population were inferred as linear. We added new conclusions regarding misspecified models based on these results.
>>> We have updated the manuscript by adding information about the new experiment in paragraph 4 of section “Orangutan demographic history” on page 7 and paragraphs 4-8 of section S2.2 on page 28 of Supplementary materials. We also added Table S17 which summarizes the histories obtained for all orangutan models without migration. Finally, we updated paragraph 6 of the "Conclusions" section on page 9 to reflect these new findings.

Inference for the Orangutan data set with models with pulse migration: This is a nice result showing that the more pulses you include, the better the estimates become. However, your main example in the main text uses the inferred migration rates. This is a poor example, because migration rates in a pulse model cannot be compared to rates in a continuous model. If migration is spread along a longer time range, then you expect the rates to decrease. So, there is no expectation of getting the same rates. You do expect, however, to get other parameters reasonably accurate. It seems like this is done with 7 pulses, but not so

much with one pulse. This should be the main the focus of the discussion of these results.

>>> We have rewritten paragraph 6 of section “Orangutan demographic history” on pages 7-8 regarding this important comment.

Results - inference of inbreeding coefficients:

When you describe the results you obtained for the cabbage data set, you say "the population size for the most recent epoch in our results is underestimated (6 vs 592 individuals) for model 1 without inbreeding and overestimated (174,960,000 vs. 215,000 individuals) for model 2 with inbreeding". The usage of under/overestimated is not ideal here, because it would imply that the original dadi estimates are more correct. You should probably simply say that they are lower/higher than estimates originally obtained by dadi. Or maybe even suggest that the original estimates were over/underestimated?

>>> Yes, thank you, we have changed it to “lower”/”higher”.

Supplementary materials:

Page 4, para2: "Figure ??" should be "Figure S1"

>>> It was fixed.

Page 4, para 4: Can you clarify what you mean by "unsupervised demographic history with structure (2, 1)"?

>>> We have included the similar description of the model with structure (2,1) as we have in the main manuscript (section S1.1 on page 4).

Page 22, para 2: "Compared to dadi and moments engines momentsLD provide slightly worse approximations for migration rates". I don't really see this in Supplementary Table S16. Estimates seem to be very similar in all methods. Am I missing anything? You make the same statement again in the STRUCT-MIG model (page 23).

>>> We agree that estimates for migration rates are very similar between all engines. We have removed those statements.

Page 22, para 4: "The best history for the ORAN-NOMIG model with restriction on population sizes is -175,106 compared to 174,309 obtained for the ORAN-STRUCT-NOMIG mod". There is a missing minus sign before the second log likelihood. You should also specify that this refers to the moments engine. Also see comment above about this result.

>>> Thank you, it was fixed.

Close
