# Supplementary material for: GADMA2: more efficient and flexible demographic inference from genetic data
Source: Gigascience. 2023 Aug 23;12:giad059. doi: 10.1093/gigascience/giad059 (PMC10445054; doi:10.1093/gigascience/giad059)
Supplement: giad059_Response_to_Reviewer_Comments_Revision_1 [file giad059_response_to_reviewer_comments_revision_1.pdf]

Author's Response To Reviewer Comments

Close

Dear Editor and Reviewers,

We appreciate the valuable feedback and suggestions provided by the reviewer #2, they definitely helped to make the manuscript better. We have incorporated the suggested revisions into both the main manuscript and the supplementary materials. The major changes in the manuscript are highlighted in blue for easy reference. A point-by-point response to the comments can be found below.

Reviewer #2:
The authors have made several substantial revisions to their manuscript, and even ran some additional experiments that provide interesting new insights. Overall, I think that the manuscript is greatly improved by this. I do list a few final comments below, but none of them are very major. One general comment is that the text added to the main manuscript (and also to the supplement) appears to require some editing to further polish it. This doesn't severely affect clarity in most cases, but the difference in quality between the original text and the added portions is noticeable.

>>> We thank the reviewer for these comments, they definitely helped a lot to make the manuscript better.

One important thing added in the revision is a more detailed explanation for how the different variants of hyperparameter sets (rounds 2-6) were compared. This is much clearer now. I appreciate the text added to the manuscript about this and figure 3, which demonstrates the speed up you get compared to the previous setup in GADMA1. However, diving into the added descriptions in the supplement (mostly section S1.2), I still get the impression that most of the comparison between different variants (runs 2-6), were done based on intuitive observations and not using very rigorous criteria. It appears as if different criteria would lead to different variants being selected. For example, if only the moments engine is considered, and the SMAC score is applied, then run 3 would have been selected, as indicated by Table S2. This appears to be the most direct extension of the initial application of SMAC. If you could run SMAC to find good values of hyperparameters without needing to
separately run it 6 times, this is likely what you would get. However, when examining different methods and other criteria, such as speed of convergence and eventual likelihood, you get other variants which appear to be better. In particular, you claim that the variant 3 leads to reduction in eventual log likelihood. This is very difficult to see from the supplementary figures (S4 and S9), so this claim is difficult for me to assess, or to weigh this reduction in likelihood with the better run times. This leads you to select from runs 2 and 4, and you eventually select run 2. This whole process seems quite tedious and not formally defined. Eventually, it ends up being one being of the weaker parts of the paper, in my opinion. It would have been nice to have clear suggestions for how to further improve the hyperparameters using SMAC, or other rigorous methodologies. I don't have a specific suggestion for an alternative approach, but I did want to point this out.

>>> Thank you very much for pointing this out one more time. We have formulated a more formal criterion based on median and quartile values of the likelihoods to compare new configurations and to select the best configuration. New figures S5 and S11 were added to supplementary materials. We have also discovered that the runs for the default configuration with moments used incorrect hyperparameter values. We reran those experiments and some results were updated.

Specific suggestions:

Introduction: paragraph 3, I suggest splitting this, now very long, paragraph into two, starting in the sentence "It was shown that…".

>>> Thank you, it was fixed.

Materials and methods: the text paragraph added toward the end of the 'Hyperparameter optimization' section should be edited to clarify several points. First, you say "within the fixed number of evaluation used to stop genetic algorithm in SMAC". This is not very clear. By "evaluation" you mean iterations of the genetic algorithms? I would also replace 'SMAC' by 'the SMAC procedure we described above'. Also, the distinction between this evaluation and what you eventually use is GADMA2 answers my question, but will not be clear to other readers. You should polish the text here to clarify that you use this measure to assess the SMAC-based selection of hyperparameters, but GADMA2 may let the genetic algorithm run more (or less) iterations, based on the criteria set by GADMA.

>>> We changed the text to provide more clarity.

Materials and methods: inference of inbreeding coefficients. I like the adjustment you do here to address Reviewer #1's concern about the number of likelihood evaluations done by GADMA and by dadi. The approach you took seems very sound. However, it did take me quite some time to understand exactly what you did. I think you can further clarify in the text for the methods and also in the caption of the tables that show these results (Tables S20, S21, S25, S26). In particular, you can say that if an average run of dadi optimization involves X evaluations, and an average run of GADMA2 optimization involves Y evaluations, then you compare a single GADMA2 run with round(Y/X) runs of dadi (where you take the run that leads to max likelihood). I realize that this is a bit detailed, but it took me time to understand this from the text and tables. In the table captions you can use similar text to explain the different number of runs you consider for dadi in each table. And one
question for clarification: you run this revised approach 100 times, right? So, in general you run dadi 100 + 100*round(Y/X) times for each of the 4 cases (the 4 supp tables mentioned above). I wanted to make sure I got it right.

>>> Thank you very much for your suggestions, it is very helpful. We have added additional sentences to clarify the procedure in the text and tables.
Yes, you understand everything correctly: we run 100 runs of single dadi optimization and 100 runs of dadi optimization with round(Y/X) repeats.

Results: in the text added to the "selection dominance rates" paragraph, can you cite studies that used the simplified approach of Williamson et al to infer dominance coefficients? This will help clarify the utility of this approach.

>>> It is a very important suggestion. However, it turned out to be very difficult to find such studies as most of the papers that cite Williamson et al are dedicated to the methods on selection but not to application of the approach. Also most of the papers that used dadi, which implements the approach from Williamson et al, are focused on inference of demographic histories without selection and dominance coefficients. It may be an indication that the method is not widely used, still our main motivation to include it was that dadi and moments have it.

Results: in the section on fruit fly demographic history. In the initial review, Reviewer #1 and I both commented about the fact that the log likelihoods for the DROS-MIG model were lower than those for DROG-NOMIG. In your response, you say that these models are nested and that this is a result of optimization failure. I noticed that Reviewer #1 also agrees with this view. However, examining the values you get in Table S6, something still doesn't sit right. The log likelihoods you get for all three methods are ~50% smaller in the MIG runs than in the NOMIG runs (across all 3 methods!). This is not a difference that I'd expect to get as a result optimization failure. I would suggest to re-examine this issue and not just write it off as a result of an optimization failure. You a very large difference also in magnitude of likelihoods in the ORANG analysis, and I don't think that this is just because of model fit. This shouldn't effect the conclusions or interpretations of the results, but the difference in log likelihoods calls for a closer examination.

>>> We argue that the difference in the likelihood values for different nested models highly depends on

the likelihood surface which is determined by the data and the model choice. Optimization challenges often arise when dealing with complex likelihood surfaces and can easily lead to great differences in likelihood values. Based on our findings with the DROG-MIG model, we propose a preliminary hypothesis that the local likelihood surface around the optimal solution without migrations is very steep. Consequently, small changes to zero migration rates can lead to substantial differences in log-likelihoods. Unfortunately, the full examination of the likelihood surface which is an interesting future work is out of the scope of this paper. We also want to highlight that there is one more example of high log-likelihood difference that is explained by the model choice. The log-likelihoods for the models without and with inbreeding for domesticated cabbage are -24,000 vs -4,000 respectively.

Results: in the section on orangutan demography. I appreciate the added experiments and detailed discussion about the issue of restricting the population size after the split. Table S17 is indeed very insightful. I also like the added result about the fact that population expansion is inferred to be linear due to model misspecification. It's especially interesting that this result is obtained across multiple likelihood engines (moments, dadi and momentsLD). Since this result is of interest, I suggest highlighting it in a separate (supplementary) table. Table S17 indicates this for moments, but it does not contain the results for the other two engines, and it contains additional information (about the restricted population size).

>>> We have added Table S18 with result demographic parameters inferred for a model with linear size change of Sumatran population. They are very similar among tested likelihood engines.

Results: inference of inbreeding coefficients. The description of the new comparison against dadi with multiple runs is not very clear. For example, the statement "single GADMA2 run provides better and more stable results than single run of dadi's optimization within 100 runs" is not very clear. You use "runs" for two different purposes. It might be better to clearly label the two types of run of dadi as the 'single run optimization' and ' multiple runs optimization', and then use "repeats" to describe the 100 replicates of the experiment. This type of ambiguity lingers throughout this paragraph and should be reworded to clarify.

>>> Thank you very much for that note. We changed the text to provide more clarity.

Results: puma demographic models. I didn't notice this in my initial review, but you use ranges when you report values in the main text. For example, "the size of the Florida population which is estimated to be 370 - 860 individuals". I now realize that these are not Cis, but rather the two point estimates inferred in model 1 and model 2. This is very confusing. I would suggest to focus on the values obtained for model 2, which received the best fit. Maybe you can specify the values for model 1 in parenthesis.

>>> Thank you, it was fixed as you suggested

Results: cabbage demographic models. In the first sentence of this section you say that GADMA2 produced models with higher likelihood. This is true only w.r.t. the "single run optimization" approach. You may wish to revise this statement by saying that it produced higher likelihoods than those obtained by Blischak et al, since those are the likelihood values that you are citing here.

>>> It was fixed

Figures S4 and S9: I don't fully understand the purpose for these figures. I understand that they mean to summarize the results you get in figs S2-3 and S7-8, but these summaries are very difficult to digest. Eventually, when you discuss these results in supp sections S1.2 and S1.3, you refer only to the mean performance of each set of hyperparameters across and across all runs and in many times across all datasets (although in section S1.3 you do differentiate datasets). So, having the full distribution described for every data set using a boxplot seems excessive and very difficult to parse. I may be missing something here, but I found it tedious to look for the main take-home message from these figures. Another thing, can you clarify the difference between the mean represented by the orange line and the mean represented by the greed triangle? Is one of them the median?

>>> Figures S4 and S10 (S9 before) are included in Supplementary materials for additional information. They provide a visual representation of the eventual log-likelihood distribution and configurations' speed.

In the revised version of the manuscript we use the median and quartiles of the log-likelihood values to compare new configurations. New figures S5 and S11 provide a summary of those comparisons, however, we prefer to have Figures S4 and S10 as supporting materials. Green triangles represent the mean values, we have changed the description.

Close
