# Supplementary material for: GADMA2: more efficient and flexible demographic inference from genetic data
Source: Gigascience. 2023 Aug 23;12:giad059. doi: 10.1093/gigascience/giad059 (PMC10445054; doi:10.1093/gigascience/giad059)
Supplement: giad059_Response_to_Reviewer_Comments_Revision_2 [file giad059_response_to_reviewer_comments_revision_2.pdf]

Author's Response To Reviewer Comments

Close

All requested revisions were made

Close
