# Supplementary material for: GADMA2: more efficient and flexible demographic inference from genetic data
Source: Gigascience. 2023 Aug 23;12:giad059. doi: 10.1093/gigascience/giad059 (PMC10445054; doi:10.1093/gigascience/giad059)
Supplement: giad059_Reviewer_1_Report_Original_Submission — Ryan Gutenkunst -- 11/7/2022 Reviewed [file giad059_reviewer_1_report_original_submission.pdf]

Reviewer Report

Title: GADMA2: more efficient and flexible demographic inference from genetic data

Version: Original Submission **Date:** 11/7/2022

Reviewer name: Ryan Gutenkunst

Reviewer Comments to Author:

In this paper, the authors present GADMA 2, an update of their population genomic inference software GADMA. The author's software serves as a driver for other population genomics software, enabling a consistent user interface and a different parameter optimization approach. GADMA 2 extends GADMA by adding two new inference engines: momi2 and momentsLD, hyperparameter optimization for the genetic algorithm, demes visualization, selection, dominance, and inbreeding modeling, and a new method for specifying model structures. In this paper, the authors show that their optimized genetic algorithm is somewhat more effective than the original hyperparameter settings. They also compare among inference engines, finding some differences in behavior. Lastly they compare with dadi itself in two models with inbreeding, finding better likelihood parameter sets than those previously published.GADMA has already found some use in the population genomics community, and GADMA 2 is a substantial update. The manuscript describes the updates in good detail and demonstrates the effectiveness of GADMA 2 on two real-world data sets. Overall, this is a strong contribution, and we have few major concerns.Major Technical Concerns:1) The authors claim to now support inference of selection and dominance. But what they support is very limited and not very biological. In particular, they currently support inferences which assume a single selection and dominance coefficient for the entire data set (as in Williamson et al. (2005) PNAS). In reality, any AFS will include sites with a variety of selection coefficients, usually summarized by a distribution of fitness effects. Since Keightley and Eyre-Walker (2007) Genetics, this has been the standard for inferring selection from the AFS. The authors should be clear about the limitations of what they have implemented.2) Figure 4 shows that optimization runs using GADMA 2 tend to find better likelihoods than bare dadi optimization runs. But the advice for using dadi or moments is to run multiple optimizations and take the best likelihood found, with some heuristic for assessing convergence. So most users would not (or at least should not) stop with the result of a single dadi optimization run. It does seem that GADMA 2 reduces the complexity of assessing convergence between multiple dadi optimization runs. But another important consideration is computational cost. (At an extreme, if each dadi run was 100 times faster than a single GADMA 2 run, then the correct comparison would be between the best of 100 dadi runs and a single GADMA 2 run.) It is not clear from the paper how the 100 GADMA 2 runs compare to the 100 dadi runs in terms of computational cost. It would be very helpful to have a table or some text describing the average computational cost (in CPU hours) of those runs.Major Writing / Presentation Concerns:1) Bottom of page 5: The authors are sharing the results of their hyperparameter optimizations from their own server, with uncertain lifetime. These results should be moved to an archival service such as Dryad.Minor Technical Concerns:1) The authors note that the DROS-MIG models had worse likelihoods than the DROS-NOMIG models. Since these are nested models, the DROS-MIG model must

mathematically have a better global optimum likelihood. It would be worth pointing out that the likelihoods they found indicate a failure of the optimization algorithms. The authors should also present the DROS-MIG model results in a supplementary table.2) The Godambe parameter uncertainties in Tables S20 and S21 are pretty extreme, sometimes 10^-13 to 10^12. This may be due to instability of the Godambe approximation versus step size. In Blischak et al. (2020) Mol Biol Evol, the authors tried several step sizes and sought consistent results between them (Tables S1-S4). We suggest the authors take that approach here.Minor Writing / Presentation Concerns:1) The author claims that "GADMA does not require model specification". However, it seems that GADMA "structure model" rather describes a different and perhaps broader way to specify demographic models rather than completely eliminates model specification.2) The authors use the term "inference engine" for the four tools GADMA 2 builds upon. But to us, the act of inference includes parameter optimization. In this case, these tools are not being used for the inference itself, but rather to calculate the (composite) likelihood of the data. Perhaps a better term would be "likelihood calculator"?3) The authors suggest engine-specific hyperparameter optimization as a future goal. But the optimal hyperparameters are also likely to be model specific. (For example, 2- versus 4-population models might benefit from different optimization regimes.) Can the authors comment on this?Writing Nitpicks1) Abstract: "optimization algorithms used to find model parameters sometimes turn out to be inefficient" â†' vague: more details on why/how they are inefficient would be helpful2) Introduction: "Inference of complex demographic histories… in the population's past." needs citation.3) Page 2: "parameter to infer, for example, all migration" is a comma splice and should be split into two sentences.4) Supplement page 4: Figure ?? reference is broken.

Level of Interest

Please indicate how interesting you found the manuscript: Choose an item.

Quality of Written English

Please indicate the quality of language in the manuscript: Choose an item.

Declaration of Competing Interests

Please complete a declaration of competing interests, considering the following questions:

- Have you in the past five years received reimbursements, fees, funding, or salary from an organisation that may in any way gain or lose financially from the publication of this manuscript, either now or in the future?
- Do you hold any stocks or shares in an organisation that may in any way gain or lose financially from the publication of this manuscript, either now or in the future?
- Do you hold or are you currently applying for any patents relating to the content of the manuscript?

- Have you received reimbursements, fees, funding, or salary from an organization that holds or has applied for patents relating to the content of the manuscript?
- Do you have any other financial competing interests?
- Do you have any non-financial competing interests in relation to this paper?

If you can answer no to all of the above, write 'I declare that I have no competing interests' below. If your reply is yes to any, please give details below.

I declare that I have no competing interests

I agree to the open peer review policy of the journal. I understand that my name will be included on my report to the authors and, if the manuscript is accepted for publication, my named report including any attachments I upload will be posted on the website along with the authors' responses. I agree for my report to be made available under an Open Access Creative Commons CC-BY license (http://creativecommons.org/licenses/by/4.0/). I understand that any comments which I do not wish to be included in my named report can be included as confidential comments to the editors, which will not be published.

Choose an item.

To further support our reviewers, we have joined with Publons, where you can gain additional credit to further highlight your hard work (see: https://publons.com/journal/530/gigascience). On publication of this paper, your review will be automatically added to Publons, you can then choose whether or not to claim your Publons credit. I understand this statement.

Yes Choose an item.
