# Supplementary material for: GADMA2: more efficient and flexible demographic inference from genetic data
Source: Gigascience. 2023 Aug 23;12:giad059. doi: 10.1093/gigascience/giad059 (PMC10445054; doi:10.1093/gigascience/giad059)
Supplement: giad059_Reviewer_2_Report_Original_Submission — Ilan Gronau -- 12/2/2022 Reviewed [file giad059_reviewer_2_report_original_submission.pdf]

Reviewer Report

Title: GADMA2: more efficient and flexible demographic inference from genetic data

Version: Original Submission **Date:** 12/2/2022

Reviewer name: Ilan Gronau

Reviewer Comments to Author:

This manuscript describes updates made to GADMA, which was published two years ago. GADMA uses likelihood-based demography inference methods as likelihood-computation engines, and replaces their generic optimization technique with a more sophisticated technique based on a genetic algorithm. The version of GADMA described in this manuscript has several important added features. It supports two additional inference engines, more flexible models, additional input and output formats, and it provides better values for the hyper-parameters used by the genetic algorithm. This is indeed a substantial improvement over the original version of GADMA. The manuscript clearly describes the different added features to GADMA, and then demonstrates them with a series of analyses. These analyses establish three main things: (1) they show that the new hyper-parameters improve performance; (2) they show how GADMA can be used to compare performance of different approaches to calculate data likelihood for demography inference; (3) showcase new features of GADMA (supporting model structure and inbreeding inference). Overall, the presentation is very clear and the results are interesting and compelling. Thus, despite being a publication about a method update, it shows substantial improvement, provides interesting new insights, and will likely lead to expansion of the user base for GADMA.The only major comment I have is about the part of the study that optimizes the hyper-parameters. The hyper-parameter optimization is a very important improvement in GADMA2. The setup for this analysis is very good, with three inference engines, four data sets used for training and six diverse data sets used for testing. However, because of complications with SMAC for discrete hyper-parameters, the analysis ends up considering six separate attempts. The comparison between the hyper-parameters produced by these six attempts is mostly done manually across data sets and inference engines. This somewhat beats the purpose of the well-designed set up. Eventually, it is very difficult for the reader to asses the expected improvement of the final suggested values of hyper-parameters (attempt 2) to the default ones. I have two comments/suggestions about this part.First, I'm wondering if there is a formal way to compare the eventual parameters of the six attempts across the four training sets. I can see why you would need to run SMAC six separate times to deal with the discrete parameters. However, why do you not use the SMAC score to compare the final settings produced by these six runs?Second, as a reader, I would like to see a single table/figure summarizing the improvement you get using whatever hyper-parameters you end up suggesting in the end compared to the default setting used in GADMA1. This should cover all the inference engines and all the data sets somehow in one coherent table/figure. Using such a table/figure, you could report improvement statistics, such as the average increase in log-likelihood, or average decrease in convergence times. These important results get lost in the many improved figures and tables.These are my main suggestions for revisions of the current version. I also have some more minor comments that the authors may wish

to consider in their revised version, which I list below.Introduction:===========para 2: the survey of demography inference methods focuses on likelihood-based methods, but there is a substantial family of Bayesian inference methods, such as MPP, Ima, and G-PhoCS. Bayesian methods solve the parameter estimation problem by Bayesian sampling. I admit that this is somewhat tangential to what GAMDA is doing, but this distinction between likelihood-based methods and Bayesian methods probably deserves a brief mention in the introduction.para 2,3: you mention a result from the original GADMA paper showing that GADMA improves on the optimization methods implemented by current demography inference methods. Readers of this paper might benefit of a brief summary of the improvement you were able to achieve using the original version of GADMA. Can you add 2-3 sentences providing the highlights of the improvement you were able to show in the first paper?para 3: The statement "GADMA separates two regular components" is not very clear. Can you rephrase to clarify?Materials and methods - Hyper-parameter optimization:==I didn't fully understand what you use for the cost function in SMAC here. Seems to me like there are two criteria: accuracy and speed. You wish the final model to be as accurate as possible (high log likelihood), but you want to obtain this result with few optimization iterations. Can you briefly describe how these two objectives are addressed in your use of SMAC? It's also not completely clear how results from different engines and different data sets are incorporated into the SMAC cost. Can you provide more details about this in the supplement?para 2: "That eliminate three combinations" should be "This eliminates three combinations".para 3: "Each attempt is running" should be "Each attempt ran"para 3: "We take 200Ã—number of parameters as the stop criteria". Can you clarify? Does this mean that you set the number of GADMA iterations to 200 times the number of demographic model parameters? Why should it be a linear function of the number of parameters? The following text explains the justification, butTable 1: I would merge Table S2 with this one (by adding the ranges of all hyper-parametres as a first column). It's important to see the ranges when examining the different selections.Materials and methods - Performance test of GADMA2 engines:===para 2: "ROS-STRUCT-NOMIG" should be "DROS-STRUCT-NOMIG" Also, "This notation could be read" - maybe replace by "This notation means" to signal that you're explaining the structure notation.Para 4 (describing comparisons for momi on Orangutan data): "ORAN-NOMIG model is compared with three …". You also consider ORAN-STRUCT-NOMIG in the momi analysis, right?Results - Performance test of GADMA2 engines:=======================================Inference for the Drosophila data set under model with migration: you mention that the models with migration obtain lower likelihoods than the models without migration. You cannot directly compare likelihoods in these two models, since the likelihood surface is not identical. So, I'm not sure that the fact that you get higher likelihoods in the models without migration is a clear enough indication for model fit. The fact that the inferred migration rates are low is a good indication for that. It also seems like despite converging to models with very low migration rates, the other parameters are inferred with higher noise. For example, the size of the European bottleneck is significantly increased in these inferences compared to that of the NOMIG. So, potentially the problem here is that more time is required for these complex models to converge.Inference for the Drosophila data set under structured model (2,1): the values inferred by moments and momentsLD appear to neatly fit the true values. However, it is not straightforward to compare an exponential increase in population size to an instantaneous increase. Maybe this can be

done by some time-averaged population size, or the average time until coalescence in the two models? This will allow you to quantify how good the two exponential models fit the true model with instantaneous increase.Inference for the Orangutan data set under structured model (2,1) without migration: you argue that a constant population size is inferred for Bor by moments and momi because of the restriction on population sizes after the split. You base this claim on a comparison between the log-likelihoods obtained in this model (STRUCT-NOMIG) and the standard model (NOMIG) in which you add this restriction. I didn't fully understand how you can conclude from this comparison that the constant size inferred for Bor is due to the restriction on the initial population size after the split. I think what you need to do to establish this is run the STRUCT model without this restriction and see that you get exponential decrease. Can you elaborate more on your rationale? A detailed explanation should appear in the supplement and a brief summary in the main text.Inference for the Orangutan data set with models with pulse migration: This is a nice result showing that the more pulses you include, the better the estimates become. However, your main example in the main text uses the inferred migration rates. This is a poor example, because migration rates in a pulse model cannot be compared to rates in a continuous model. If migration is spread along a longer time range, then you expect the rates to decrease. So, there is no expectation of getting the same rates. You do expect, however, to get other parameters reasonably accurate. It seems like this is done with 7 pulses, but not so much with one pulse. This should be the main the focus of the discussion of these results.Results - inference of inbreeding coefficients:=======================================When you describe the results you obtained for the cabbage data set, you say "the population size for the most recent epoch in our results is underestimated (6 vs 592 individuals) for model 1 without inbreeding and overestimated (174,960,000 vs. 215,000 individuals) for model 2 with inbreeding". The usage of under/overestimated is not ideal here, because it would imply that the original dadi estimates are more correct. You should probably simply say that they are lower/higher than estimates originally obtained by dadi. Or maybe even suggest that the original estimates were over/underestimated?Supplementary materials:=====================Page 4, para2: "Figure ??" should be "Figure S1"Page 4, para 4: Can you clarify what you mean by "unsupervised demographic history with structure (2, 1)"?Page 22, para 2: "Compared to dadi and moments engines momentsLD provide slightly worse approximations for migration rates". I don't really see this in Supplementary Table S16. Estimates seem to be very similar in all methods. Am I missing anything? You make the same statement again in the STRUCT-MIG model (page 23).Page 22, para 4: "The best history for the ORAN-NOMIG model with restriction on population sizes is -175,106 compared to 174,309 obtained for the ORAN-STRUCT-NOMIG mod". There is a missing minus sign before the second log likelihood. You should also specify that this refers to the moments engine. Also see comment above about this result.

Level of Interest

Please indicate how interesting you found the manuscript: Choose an item.

Quality of Written English

Please indicate the quality of language in the manuscript: Choose an item.

Declaration of Competing Interests

Please complete a declaration of competing interests, considering the following questions:

- Have you in the past five years received reimbursements, fees, funding, or salary from an organisation that may in any way gain or lose financially from the publication of this manuscript, either now or in the future?
- Do you hold any stocks or shares in an organisation that may in any way gain or lose financially from the publication of this manuscript, either now or in the future?
- Do you hold or are you currently applying for any patents relating to the content of the manuscript?
- Have you received reimbursements, fees, funding, or salary from an organization that holds or has applied for patents relating to the content of the manuscript?
- Do you have any other financial competing interests?
- Do you have any non-financial competing interests in relation to this paper?

If you can answer no to all of the above, write 'I declare that I have no competing interests' below. If your reply is yes to any, please give details below.

I declare that I have no competing interests

I agree to the open peer review policy of the journal. I understand that my name will be included on my report to the authors and, if the manuscript is accepted for publication, my named report including any attachments I upload will be posted on the website along with the authors' responses. I agree for my report to be made available under an Open Access Creative Commons CC-BY license (http://creativecommons.org/licenses/by/4.0/). I understand that any comments which I do not wish to be included in my named report can be included as confidential comments to the editors, which will not be published.

Choose an item.

To further support our reviewers, we have joined with Publons, where you can gain additional credit to further highlight your hard work (see: https://publons.com/journal/530/gigascience). On publication of this paper, your review will be automatically added to Publons, you can then choose whether or not to claim your Publons credit. I understand this statement.

Yes Choose an item.
